# Tumor antigens and immune subtypes of glioblastoma: the fundamentals of mRNA vaccine and individualized immunotherapy development

**DOI:** 10.1186/s40537-022-00643-x

**Published:** 2022-07-14

**Authors:** Changwu Wu, Chaoying Qin, Wenyong Long, Xiangyu Wang, Kai Xiao, Qing Liu

**Affiliations:** 1 Department of Neurosurgery, Xiangya Hospital, Central-South University, 87 Xiangya Road, Changsha, 410008 Hunan People’s Republic of China; 2 Institute of Skull Base Surgery and Neuro-Oncology at Hunan, Changsha, China

**Keywords:** Glioblastoma, mRNA vaccines, Immune subtypes, Tumor microenvironment, Individualized immunotherapy

## Abstract

**Purpose:**

Glioblastoma (GBM) is the most common primary brain tumor in adults and is notorious for its lethality. Given its limited therapeutic measures and high heterogeneity, the development of new individualized therapies is important. mRNA vaccines have exhibited promising performance in a variety of solid tumors, those designed for glioblastoma (GBM) need further development. The aim of this study is to explore tumor antigens for the development of mRNA vaccines against GBM and to identify potential immune subtypes of GBM to identify the patients suitable for different immunotherapies.

**Methods:**

RNA-seq data and the clinical information of 143 GBM patients was extracted from the TCGA database; microarray data and the clinical information of 181 GBM patients was obtained from the REMBRANDT cohort. A GBM immunotherapy cohort of 17 patients was obtained from a previous literature. GEPIA2, cBioPortal, and TIMER2 were used to identify the potential tumor antigens. Immune subtypes and gene modules were identified using consensus clustering; immune landscape was constructed using graph-learning-based dimensionality reduction analysis.

**Results:**

Nine potential tumor antigens associated with poor prognosis and infiltration of antigen-presenting cells were identified in GBM: ADAMTSL4, COL6A1, CTSL, CYTH4, EGFLAM, LILRB2, MPZL2, SAA2, and LSP1. Four robust immune subtypes and seven functional gene modules were identified and validated in an independent cohort. Immune subtypes had different cellular and molecular characteristics, with IS1, an immune cold phenotype; IS2, an immune hot and immunosuppressive phenotype; IS3, a relatively immune cold phenotype, second only to IS1; IS4, having a moderate tumor immune microenvironment. Immune landscape revealed the immune distribution of the GBM patients. Additionally, the potential value of immune subtypes for individualized immunotherapy was demonstrated in a GBM immunotherapy cohort.

**Conclusions:**

ADAMTSL4, COL6A1, CTSL, CYTH4, EGFLAM, LILRB2, MPZL2, SAA2, and LSP1 are the candidate tumor antigens for mRNA vaccine development in GBM, and IS1 GBM patients are best suited for mRNA vaccination, IS2 patients are best suited for immune checkpoint inhibitor. This study provides a theoretical framework for GBM mRNA vaccine development and individualized immunotherapy strategies.

**Supplementary Information:**

The online version contains supplementary material available at 10.1186/s40537-022-00643-x.

## Introduction

Glioblastoma (GBM), a World Health Organization grade 4 glioma, is notorious for its highly lethal and refractory nature. Compared with low-grade glioma, GBM develops faster and has a worse prognosis [1, 2]. GBM is usually located in the subcortex, has an infiltrative growth, and often invades multiple cerebral lobes and deep structures; thus, it is extremely difficult to operate on [3]. Despite the current standard of care, with multimodal therapy including surgery, temozolomide chemotherapy, and radiotherapy, the median overall survival (OS) of GBM patients is only about 19 months, and postoperative recurrence is almost unavoidable [4–6]. Hence, there is an urgent need to devise new treatment strategies to improve the management of GBM patients.

The role of immunotherapy in GBM has garnered attention because of the significant clinical benefits achieved with this treatment modality in a variety of malignancies, including melanoma and hepatocellular carcinoma [7–10]. However, current attempts to use immunotherapy for GBM have resulted in limited success. For example, while immune checkpoint inhibitor (ICI) monotherapies, including anti-PD-1 and anti-CTLA4, have been used successfully to treat a variety of cancers [11, 12], no significant efficacy has been seen in GBM so far [13, 14]. A recent study showed that T cells expressing multiple immune checkpoints in GBM were more dysfunctional than the T cells expressing only the PD-1 checkpoint [15]. This suggests that combination therapies targeting multiple immune checkpoints may be more effective in GBM compared to ICI monotherapy. In addition to ICI therapy, use of tumor vaccines is another therapy that has attracted great interest in GBM. There are four main types of tumor vaccines being developed: peptide vaccines, tumor cell or immune cell vaccines, viral vector vaccines, and nucleic acid (DNA or RNA) vaccines [16]. Tumor vaccine research in GBM is mainly focused on peptide vaccines. Among these, a peptide vaccine targeting EGFRvIII (rindopepimut) lead to slightly mitigated GBM progression in phase II clinical studies [17–19]. Although the vaccine resulted in only a few months of clinical benefit, it indicates the vast potential for tumor vaccines in the treatment of GBM. In addition, another peptide vaccine, ICT-107, for GBM showed some efficacy in a phase II clinical trial [20, 21] in which six peptides of the vaccine were significantly overexpressed in GBM. Unfortunately, the phase III trial of this vaccine was terminated due to problems in funding. Unlike peptide vaccines, nucleic acid vaccines can encode full-length tumor antigens, are not restricted by human leukocyte antigen (HLA) type, and are more likely to stimulate a broad T-cell response [22]. In addition, nucleic acid vaccines can deliver multiple antigens including both tumor-associated antigens (TAAs) and tumor-specific antigens (TSAs), making them more resistant to the development of drug resistance [23]. Additionally, the production of nucleic acid vaccines can be more rapid and cost-effective compared to peptide vaccine production, which is time-consuming and labor-intensive [24, 25]. Among the nucleic acid vaccines, messenger RNA (mRNA) vaccines have very significant advantages over DNA vaccines, which include the advantage of expression rate and magnitude and the safety advantage of not integrating into the cellular genome, thereby preventing insertional mutations [22, 25, 26]. More importantly, the ease with which mRNA sequences can be designed and modified allows them to encode any pathological antigen easily and rapidly, which is particularly advantageous in the individualized treatment of tumors, especially actual the pandemic period [25]. Although mRNA vaccines are relatively unstable structurally and generally require cold storage below 0 °C and for short periods of time [27], the widespread use of the two mRNA vaccines (BioNTech and Moderna) in the COVID-19 pandemic and their impressive efficiency still further prove their superiority [28, 29]. Until now, the efficacy of mRNA vaccines in a variety of solid tumors has been promising [23]. For example, in the clinical trials of advanced non-small cell lung cancer and prostate cancer, mRNA vaccines induced specific immune responses and improved patient prognosis [30, 31]. However, the mRNA vaccine designed for GBM needs to be developed further, and the screening of vaccine-sensitive patients based on their immune characteristics is necessary.

The aim of this study was to explore tumor antigens for the development of anti-GBM mRNA vaccines and to identify immunophenotypes that could be used to screen the GBM patients suitable for different immunotherapies including mRNA vaccines.

## Methods

### Patients and datasets

RNA-seq data for the 143 GBM patients in The Cancer Genome Atlas (TCGA) discovery cohort and the clinical information were obtained from the University of California, Santa Cruz, Xena Functional Genomics Explorer (https://xenabrowser.net/datapages/) (Additional file 1: Table S1 and Additional file 2: Table S2). RNA-seq data was normalized by log2(x + 1) transformation. The normalized expression matrix and the clinical information of the 181 GBM cases in the REMBRANDT microarray validation cohort were obtained from GlioVis (http://gliovis.bioinfo.cnio.es/) (Additional file 1: Table S1) [32]. The normalized RNA-seq data and clinical information for 17 GBM patients in the PD-1 inhibitors (nivolumab or pembrolizumab) cohort were obtained from the study by Zhao et al. (Additional file 1: Table S1) [33]. All data were obtained from the corresponding author or supplemental material of this publication. All cases in our study are adults and cases without complete clinical information were excluded, and only the genes with non-zero expression levels in > 50% of the samples were retained.

### Identification of tumor antigens

The cBioPortal (https://www.cbioportal.org/) tool integrates raw data from multiple cohorts, including TCGA cohort [34]. In this study, we used the cBioPortal tool to visualize genetic alterations in GBM patients and to screen the mutant genes.

In this study, differentially expressed genes in GBM patients were identified by ANOVA using the “Differential Genes” module of Gene Expression Profiling Interactive Analysis version 2 (GEPIA2, http://gepia2.cancer-pku.cn/#index) tool [35], with parameters set to |log_2_FC| value > 1 and q < 0.01. In addition, the OS and relapse-free survival (RFS) of GBM patients were evaluated using the Kaplan–Meier “survival analysis” module in GEPIA2. GBM cases were divided into high and low expression groups based on the median value of selected tumor antigen expression, Kaplan–Meier curves were obtained, and log-rank test p values and hazard ratios were calculated. Statistical significance was set at p < 0.05.

Tumor Immune Estimation Resource (TIMER, https://cistrome.shinyapps.io/timer/) is a web server for the comprehensive analysis of tumor-infiltrating immune cells, among them, dendritic cells include resting dendritic cells and activated dendritic cells, and macrophages include monocytes, macrophage M0, macrophage M1 and macrophage M2 [36]. In this study, the correlation between identified tumor antigen expression and antigen-presenting cell (APC) infiltration was evaluated using a purity-adjusted partial Spearman’s correlation analysis based on the TIMER tool. Statistical significance was set at p < 0.05.

### Identification and validation of immune subtypes

Firstly, we extracted 2348 immune-related genes from the scientific literature and the ImmPort database (Additional file 2: Table S3) [37, 38]; this comprehensive gene set included the genes responsible for various immune processes such as antigen processing and presentation, production of chemokines, etc. Subsequently, based on the immune-related gene expression profiles, unsupervised consensus clustering was performed using the partition around medoids (PAM) algorithm to identify robust clusters of GBM. A total of 1000 bootstraps were executed and each bootstrap resampled 80% of the patients in the TCGA cohort. The maximum number of clusters was 10, and K values were evaluated using a consensus cumulative distribution function and consensus heatmap [39].

The verification of the immune subtypes (ISs) identified in the TCGA cohort was done as follows: we first determined the immune-related genes shared by the TCGA and REMBRANDT cohorts and calculated the centroid of each IS of the shared immune-related genes in the TCGA cohort; next, we trained a PAM-based classifier in the TCGA cohort to predict IS for cases in the validation cohort. Specifically, each sample in the REMBRANDT cohort was assigned to an IS, the centroid of which has the highest Pearson correlation with the given sample. An intra-group proportion (IGP) analysis was performed to assess the reproducibility and similarity of IS between the TCGA and REMBRANDT cohorts [40]. Meanwhile, we also used the PAM-based classifier to identify IS in the PD-1 inhibitor cohort. The R package “limma” was used to identify differentially expressed genes (DEGs) between pre- and post-immunotherapy samples, and Kyoto encyclopedia of genes and genomes (KEGG) pathway analysis and Gene ontology (GO) biological process enrichment analysis of DEGs were performed using R package “clusterProfiler”.

### Identification of immune gene modules

Identification of robust immune gene modules (GMs) was done by applying consensus clustering using the same settings and parameters that were employed for IS identification in the discovery cohort. Next, the biological functions of each GM were annotated. The genes in each GM were annotated in terms of GO biological processes by R package “clusterProfiler”. GMs in the validation cohort were defined using the same gene arrangement as in the discovery cohort. Scores for GM (GM scores) were defined as the average expression levels of all the genes in a particular module.

### Evaluation of molecular and cellular characteristics related to immune subtypes

Single-sample gene set enrichment analysis (ssGSEA) was performed using the R package “GSVR” to quantify the abundance of 28 immune cell infiltration in each sample [41]. In addition, the relationship between IS and the 56 previously reported immune-related molecular and cellular features was assessed [42].

### Analysis of the immune landscape

To further reveal the intrinsic structure of IS and the distribution of individual cases, we performed a dimensionality reduction analysis based on a graph-learning-based approach. Discriminative dimensionality reduction with trees (DDRTree) was performed using the reduceDimension function in the R package “Monocle” with the maximum component setting of two, and was visualized using the plot cell trajectory function [43].

### Establishment of immune proteins interaction network

Protein–protein interaction (PPI) networks were constructed using genes from the GM by employing the STRING tool (https://string-db.org/) [44]. Subsequently, hub genes in the PPI networks were obtained using the cytoHubba plugin in the Cytoscape software [45].

## Results

### Identification of potential tumor antigens in GBM

**Fig. 1:**
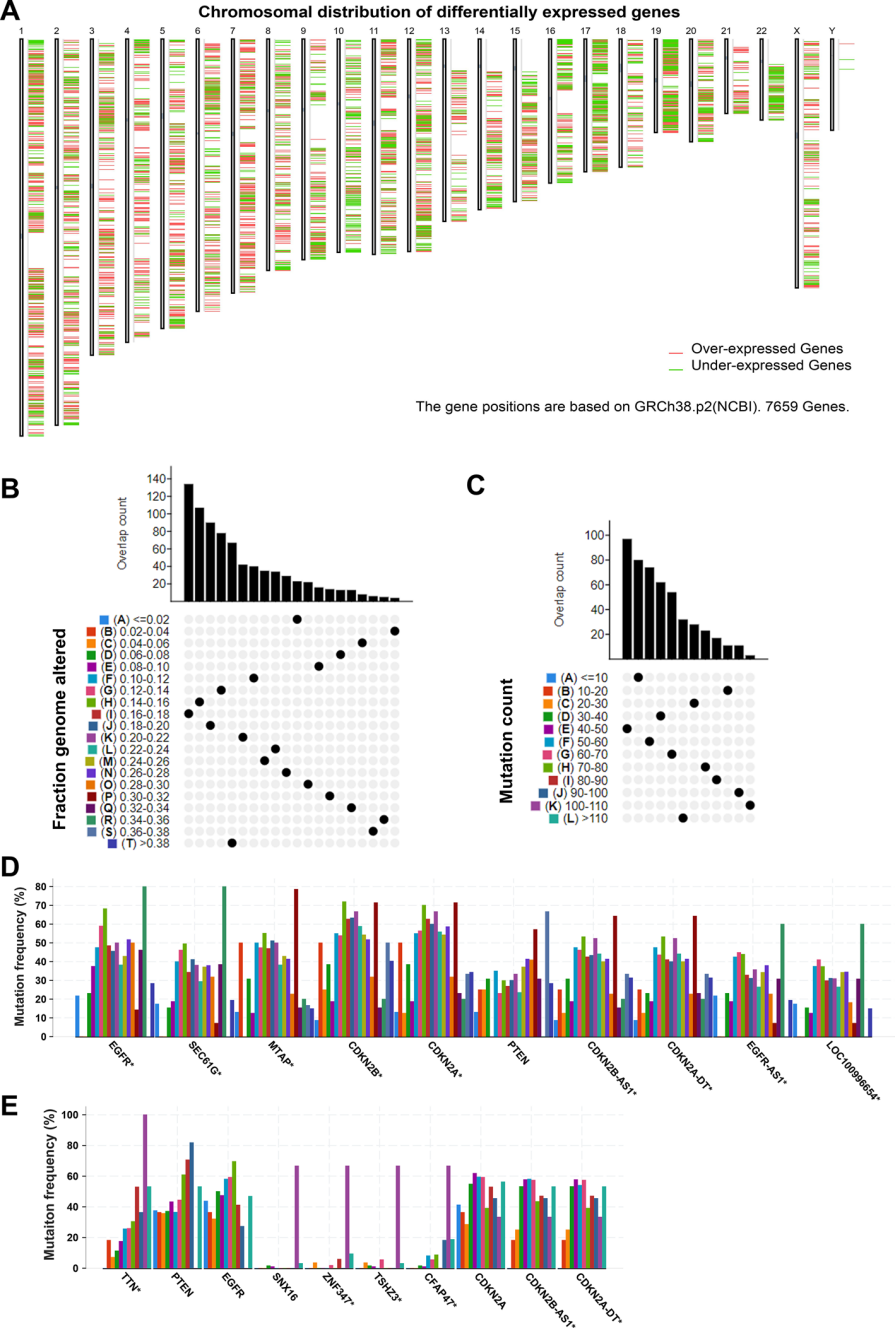
Identification of potential tumor antigens in GBM. **a** The chromosomal distribution of up- and down-regulated genes in GBM. **b** Samples overlapping in the altered genome fraction group. **c** Samples overlapping in the mutation count group. **d** Genes with highest frequency in the fraction genome altered group. **e** Genes with highest frequency in the mutation count group

To explore the potential tumor antigens in GBM, firstly, the aberrantly expressed genes were identified from the GBM gene expression profile and 5221 overexpressed genes that may encode TAAs were filtered (Fig. 1A). Subsequently, based on the fractional genomic alternations and mutation counts of individual samples, 6,585 mutation genes potentially encoding TSAs were filtered (Fig. 1B, C). Notably, most patients had low fractional genomic alterations and mutation counts, suggesting low immunogenicity of GBM (Fig. 1B, C). In Fig. 1D, E, the top 10 genes in the fractional genomic alteration and mutation count groups in terms of alteration frequency are shown, respectively. It is worth noting that epidermal growth factor receptor, phosphatase and tensin homolog (PTEN), cyclin dependent kinase inhibitor 2A, and CDKN2B antisense RNA 1 had a high alteration frequency in both the groups (Fig. 1D, E). Considering the intersection of the overexpression group and the mutation group, 1332 overexpressed and frequently mutated genes were obtained.

**Fig. 2:**
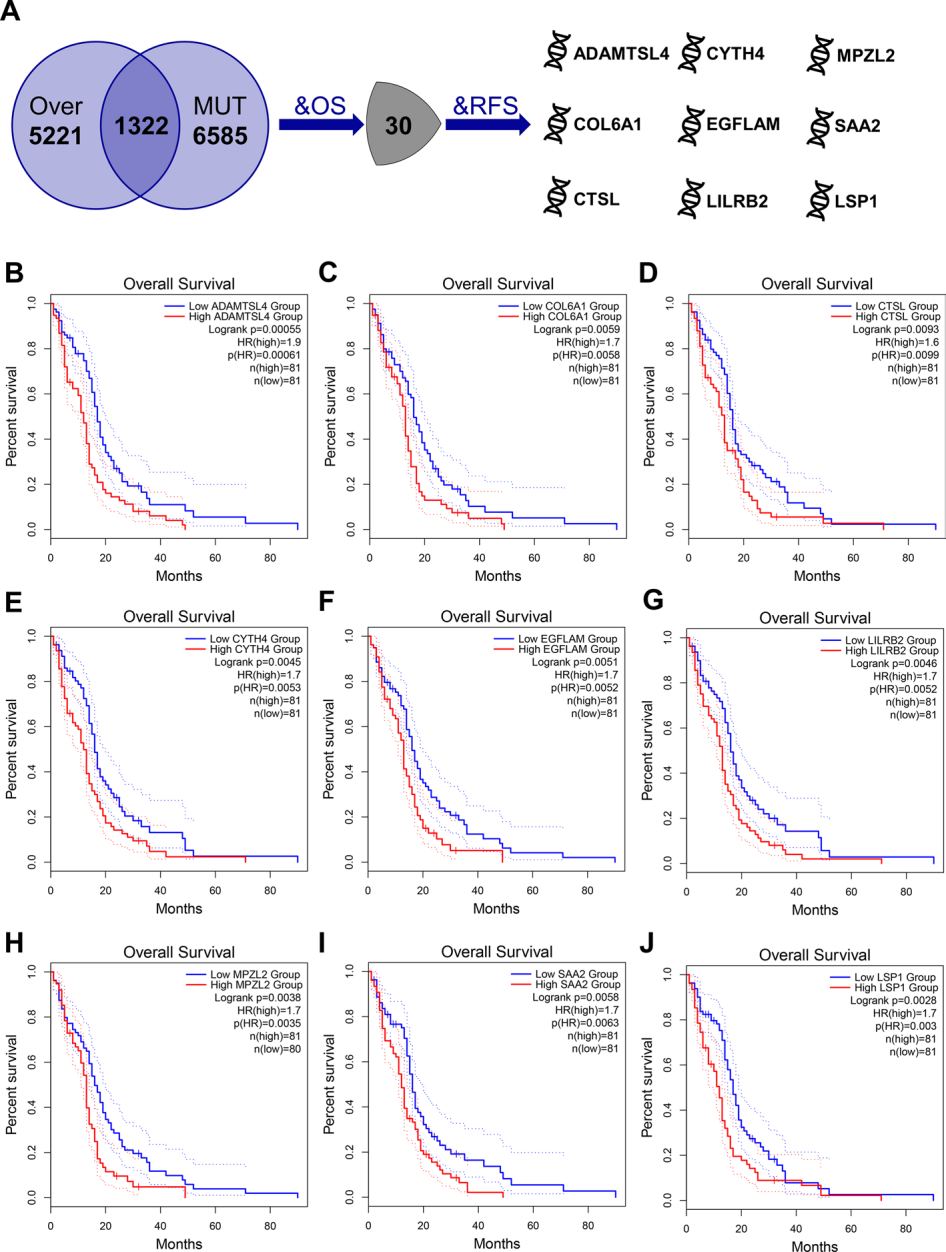
Identification of tumor antigens associated with GBM prognosis. **a** Potential tumor antigens (total 1332) with both overexpression and mutation, and tumor antigens significantly associated with OS and RFS (total 9 candidates). Kaplan–Meier curves comparing OS for groups with different expression of ADAMTSL4 (**b**), COL6A1 (**c**), CTSL (**d**), CYTH4 (**e**), EGFLAM (**f**), LILRB2 (**g**), MPZL2 (**h**), SAA2 (**i**), and LSP1 (**j**) in GBM. Red lines represented high gene expression, blue represented low gene expression

Further, identification of tumor antigens that may play a critical role in the progression of GBM and can be used in mRNA vaccine development was achieved by screening 30 candidate genes that were closely associated with OS of GBM among the above mentioned genes, nine of which were significantly associated with RFS (Fig. 2A). As shown in the Fig. 2B–J, patients with high expression of ADAMTS-like 4 (ADAMTSL4), collagen type VI alpha 1 chain (COL6A1), cathepsin L (CTSL), cytohesin 4 (CYTH4), EGF like, fibronectin type III and laminin G domains (EGFLAM), leukocyte immunoglobulin-like receptor B2 (LILRB2), myelin protein zero like 2 (MPZL2), serum amyloid A2 (SAA2), and lymphocyte specific protein 1 (LSP1) in GBM had significantly poor OS and RFS (Additional file 1: Figure S1). In summary, nine candidate genes that undergo overexpression and mutation are highly relevant to the progression of GBM. It was also found that the expression of these nine candidate genes in GBM had significant positive correlation with dendritic cell infiltration, and the expression of SAA2 was also positively correlated with macrophage infiltration (Additional file 1: Figure S2A–I). This implies that these nine tumor antigens may be directly processed and presented by the APCs to induce an immune response, making them promising candidates for the development of GBM mRNA vaccines. In addition, it is noteworthy that by stepwise regression we found that LSP1 and ADAMTSL4 could constitute a two-gene signature. The risk score based on this gene signature can predict the prognosis of GBM patients (Additional file 1: Figure S3A–C).

### Identification of potential immune subtypes in GBM

**Fig. 3:**
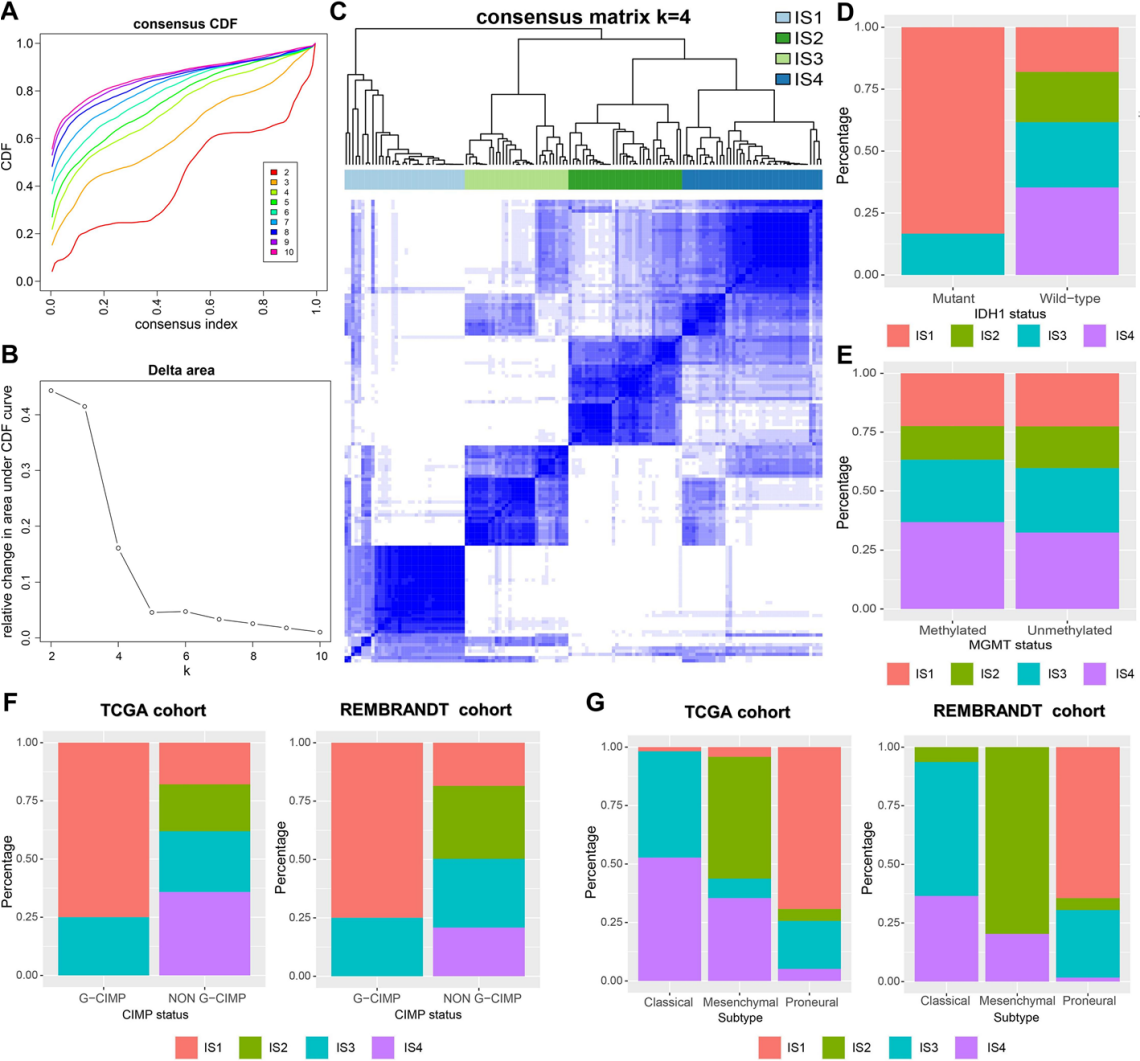
Identification of potential immune subtypes in GBM. Cumulative distribution function curve (**a**), delta area curve (**b**) and consensus heatmap (**c**) based on immune-related gene expression profile in the TCGA cohort. **d** The distribution of immune subtypes in GBM patients with different IDH1 status in TCGA cohort. **e** The distribution of immune subtypes in GBM patients with different MGMT status in the TCGA cohort. **f** The distribution of immune subtypes in GBM patients with different G-CIMP status in the TCGA cohort and the REMBRANDT cohort. **g** The distribution of immune subtypes in GBM patients with different molecular subtype in the TCGA cohort and the REMBRANDT cohort

Identification of potential immune subtypes in GBM for screening of patients suitable for mRNA vaccination was achieved by clustering the 143 GBM cases from the TCGA cohort using consensus clustering based on immune-related gene expression profile. After analyzing the consensus cumulative distribution function, delta area, and consensus heatmap, we chose K = 4 and identified four ISs in GBM, namely IS1–IS4 (Fig. 3A–C). GBM cases in the REMBRANDT validation cohort were also assigned to the four ISs. In addition, ISs were found to have no significant correlation with OS in either of the cohorts (Additional file 1: Figure S4). Given the widely known clinical relevance of isocitrate dehydrogenase [NADP (+)] 1 (IDH1) mutation, O-6-methylguanine-DNA methyltransferase (MGMT) methylation and cytosine-phosphate-guanine (CpG) island methylator phenotype (CIMP) status in GBM [2], we also explored the distribution of ISs in different IDH1 mutation, MGMT methylation and CIMP states. Notably, patients with IDH1 mutant phenotype belonged mainly to the IS1, followed by the IS3 (Fig. 3D). IS1–IS4 were evenly distributed in both MGMT-methylated and unmethylated patients (Fig. 3E). The distribution of ISs in patients with different CIMP status showed that all the glioma-CIMP (G-CIMP) patients were identified as IS1 and IS3, whereas, the non-G-CIMP patients were found to have all four ISs; this result was also validated in the REMBRANDT cohort (Fig. 3F). In addition, in both the TCGA cohort and the REMBRANDT cohort, patients with the classical subtype were predominantly IS3 and IS4, patients with the mesenchymal subtype were predominantly IS2 and IS4, and patients with the proneural subtype were mainly IS1 and IS3 (Fig. 3G). The above results implied reproducibility of ISs in the discovery and validation cohorts, while the IGP analysis further confirmed moderate to good agreement between the two cohorts (Additional file 1: Table S4).

### The relationship between immune subtypes and mutational status

It is well known that tumor mutational burden (TMB) and the number of somatic mutations are closely related to the effect of immunotherapy [46]. Therefore, TMB and mutations in IS1-IS4 were analyzed based on mutation data from the varscan-processed mutation dataset of the TCGA cohort. As shown in Additional file 1: Figure S5A, B, there was no significant difference in the TMB and the number of mutated genes in the four ISs. This demonstrated that the amount of tumor antigen encoded by the mutated genes did not differ among the ISs, and overall GBM has a relatively low TMB. In Additional file 1: Figure S5C, we showed the top 10 most frequently mutated genes in the four ISs, including PTEN.

### The relationship between immune subtypes and immunomodulators

**Fig. 4:**
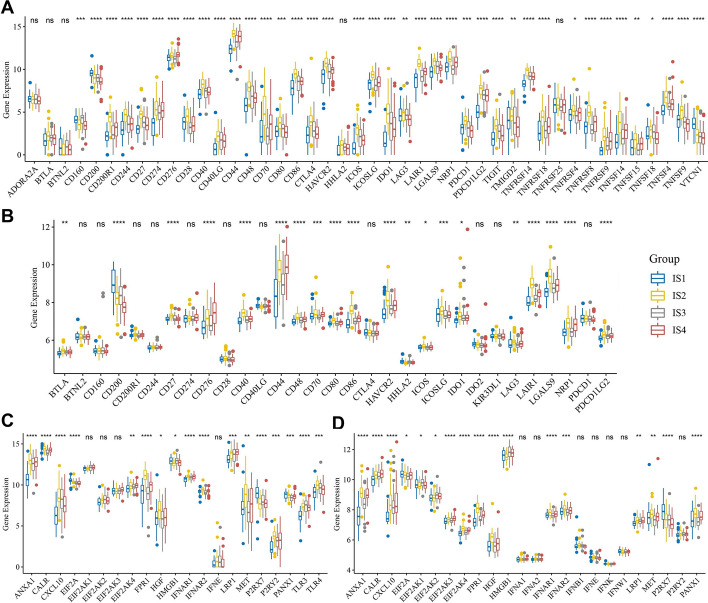
Association between immune subtypes and immunomodulators in GBM. Differences in expression levels of ICP-related genes among GBM immune subtypes in the TCGA cohort (**a**) and the REMBRANDT cohort (**b**). Differences in expression levels of ICD-related genes among GBM immune subtypes in the TCGA cohort (**c**) and the REMBRANDT cohort (**d**). *p < 0.05, **p < 0.01, ***p < 0.001, ****p < 0.0001

Immune checkpoints (ICPs) and immunogenic cell death (ICD) modulators play an important role in tumor immunity and may influence the selection of different immunotherapies including mRNA vaccines [47]. In the present study, we detected 44 expressed ICP-related genes from the TCGA cohort; 39 (88.6%) of these had significant expression differences among the ISs (Fig. 4A). We detected 31 expressed ICP-related genes from the REMBRANDT cohort, and 20 (64.5%) of these were differentially expressed among the ISs (Fig. 4B). Notably, in the TCGA cohort, patients with IS2 showed significantly higher expression of almost all the ICP-related genes, including CD274 (also known as PD-L1) and CTLA4. In contrast, in patients with IS1, most ICP-related genes showed low levels of expression. Increased expression of most of the ICP-related genes in IS2 was also observed in the REMBRANDT cohort. In addition, we also detected 21 expressed ICD-related genes in the TCGA cohort, and 17 (81.0%) of them were differentially expressed among the ISs (Fig. 4C). For example, EIF2A, HMGB1, P2RX7, and PANX1 were significantly highly expressed in IS1. In the REMBRANDT cohort, 24 ICD-related genes were detected, of which, 17 (70.8%) were differentially expressed among the ISs (Fig. 4D). EIF2A and P2RX7 were also significantly highly expressed in IS1 in the REMBRANDT cohort. Therefore, different ISs reflect different expression levels of ICPs and ICD modulators, contributing to the selection of patients suitable for mRNA vaccination.

### Molecular and cellular characteristics of immune subtypes

**Fig. 5:**
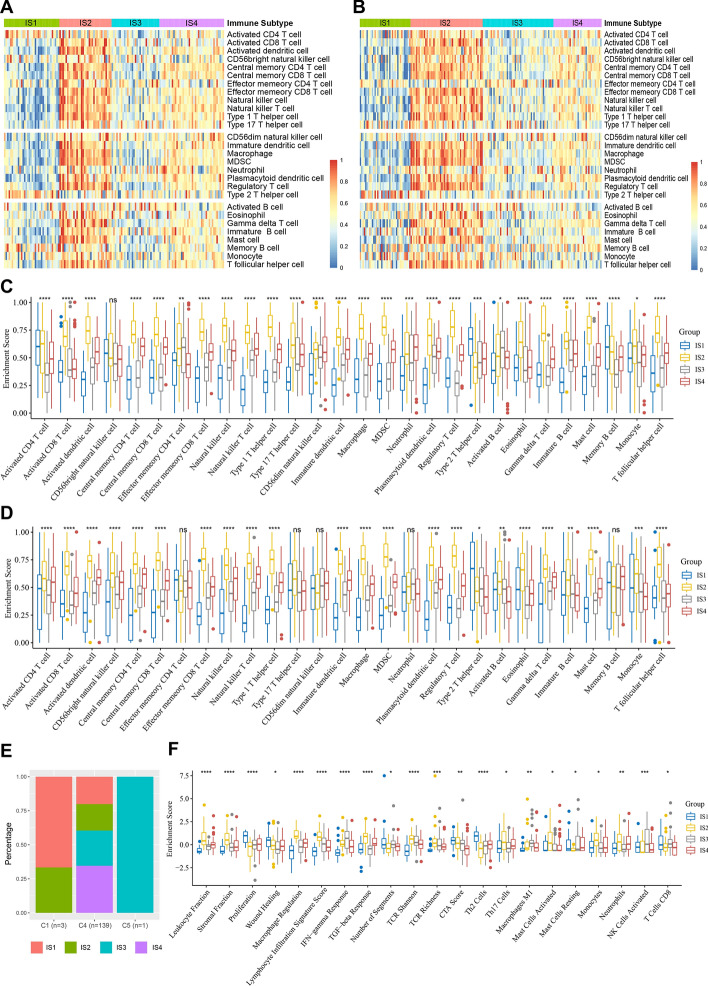
Cellular and molecular characteristic of immune subtypes in GBM. Heatmaps of 28 previously reported immune cell signatures scores among GBM immune subtypes in the TCGA cohort (**a**) and the REMBRANDT cohort (**b**). Differences of 28 immune cell signatures scores among GBM immune subtypes in the TCGA cohort (**c**) and the REMBRANDT cohort (**d**). **e** The distribution of GBM four immune subtypes in the pan-cancer immune subtypes. **f** 21 immune-related molecular signatures with significant differences among GBM immune subtypes. *p < 0.05, **p < 0.01, ***p < 0.001, ****p < 0.0001

Since the effectiveness of immunotherapy is linked to the infiltration of immune cells in the tumor microenvironment (TME), we scored the enrichment of 28 immune cells using the ssGSEA method [41]. As shown in Fig. 5 A, B, the 28 immune cells were divided into four clusters based on ISs in both the cohorts, and the components of immune cells were significantly different among the ISs (Fig. 5C, D). For example, IS2 in TCGA cohort had the highest scores for almost all immune-stimulating cells such as activated CD8^+^ T cells, activated dendritic cells, and natural killer cells, and also for almost all immunosuppressive cells such as myeloid-derived suppressor cells (MDSCs) and regulatory T cells. Contrary to IS2, IS1 had the lowest scores for most of the immune cells, while IS3 has the second lowest score after IS1 (Fig. 5A, C). Similar results were observed in the REMBRANDT cohort (Fig. 5B, D). This suggests an immune hot but strongly immunosuppressive TME in IS2, whereas IS1 is an immune cold phenotype, IS3 is a relatively cold phenotype, and IS4 has a moderate and complex TME.

In the previous studies, Thorsson et al. developed a pan-cancer immunophenotyping method, which divided 33 cancers, including GBM, into six immunotypes (C1–C6) with different characteristics [42]. Here, we explored pan-cancer immunophenotyping in relation to the four ISs in our study. As shown in Fig. 5E, almost all the GBM samples belonged to the C4 immunotype, and of the only three C1 samples, two were IS1 and one was IS2; while the only C5 sample belonged to IS3. This indicates that the TME of GBM is very different from that of other tumors, and the immunophenotyping method developed in our study may be more applicable to GBM and is complementary to the classifications mentioned in the previous studies. Furthermore, the relationship between ISs and the 56 previously reported immune-related molecular signatures was examined, 21 of which differed significantly among the ISs (Fig. 5F). IS2 had the highest scores in leukocyte fraction, lymphocyte infiltration, and Th17 cells, along with the highest scores in stromal fraction, TGF-β response, macrophage regulation, and mast cell activated signatures, indicating an immune hot and immunosuppressive phenotype. In contrast, the lowest leukocyte fraction, lymphocyte infiltration, macrophage M1, IFN-γ response, stromal fraction, macrophage regulation, and TGF-β response scores in IS1 reflected an immunological cold phenotype. IS3 was characterized by a relatively low leukocyte fraction, Th17 cells, stromal fraction, TGF-β response, macrophage regulation, neutrophils, and the highest IFN-γ response, macrophage M1, and NK cell activation, suggesting a relatively cold phenotype with some immune activation. IS4 showed moderate immune infiltration, suggesting a complex TME. These results are consistent with the previous results. In summary, ISs can reflect the immune status of GBM patients and are promising immunotherapy biomarkers. IS1 and IS3, which have an immunologically cold or relatively cold phenotype, could be potential mRNA vaccine-susceptible patients, while IS2 could be suitable for ICI threapy.

### The immune landscape of GBM

**Fig. 6:**
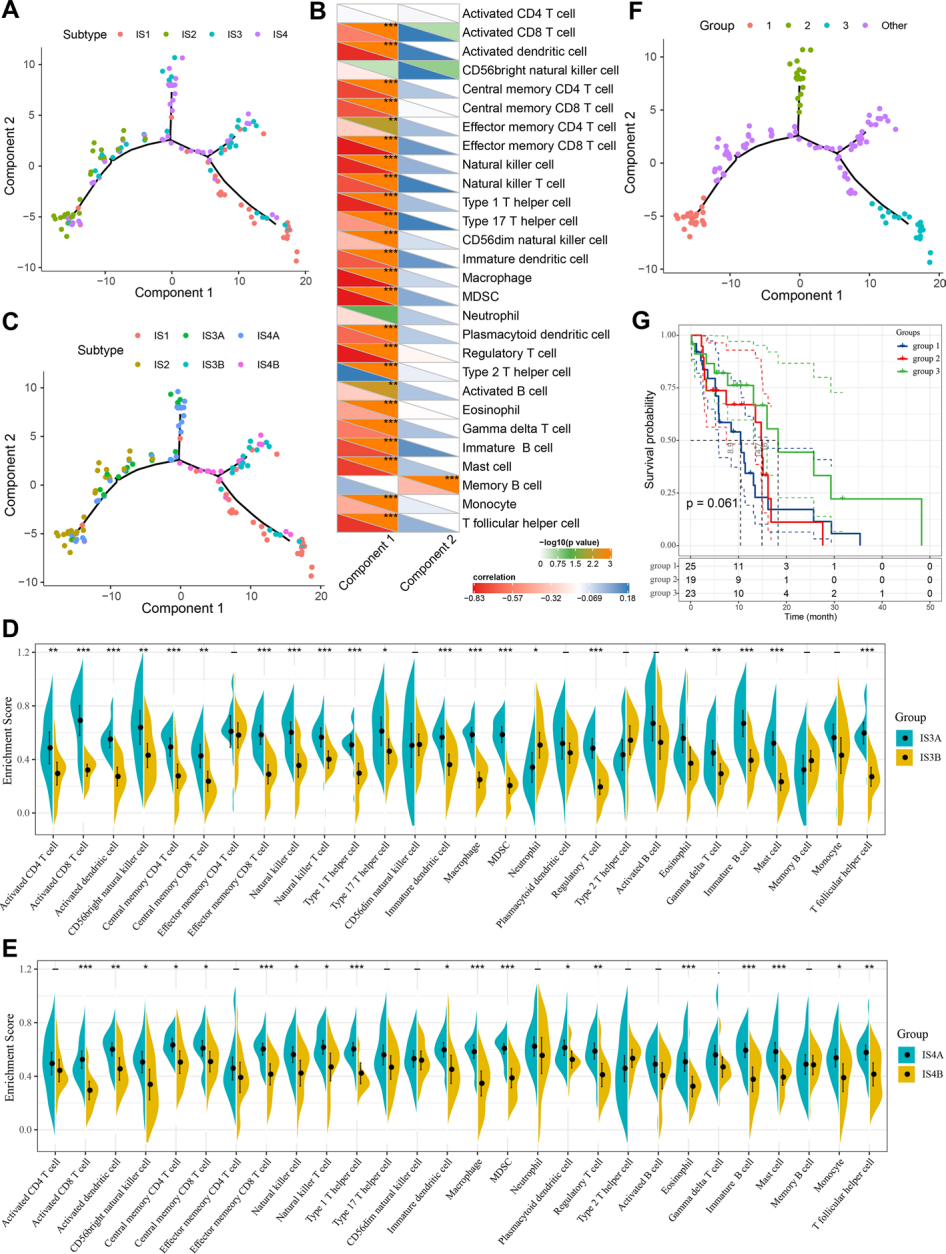
The immune landscape of GBM. **a** The immune landscape of GBM. Each dot represents a patient, and different colors represent different immune subtypes. The horizontal axis represents the principal component 1, and the vertical axis represents the principal component 2. **b** Correlation between principal component 1/2 and 28 immune cell enrichment scores. **c** Immune landscape of the subgroups of GBM immune subtypes. Differences of 28 immune cells enrichment scores in the subgroups of IS3 (**d**) and IS4 (**e**). Immune landscape of samples from three extreme locations (**f**) and their prognostic status (**g**). ^−^*p* ≥ 0.1, ^·^*p* < 0.1, *p < 0.05, **p < 0.01, ***p < 0.001

To visualize the immune distribution of each patient to complement the previously defined ISs, the immune landscape of GBM was constructed based on the immune gene expression profiles of individual patients and the graph-learning-based dimensionality reduction analysis. Each point represented a patient, the horizontal axis was principal component 1 based on the immune gene expression profile and the vertical axis was principal component 2 (Fig. 6A). Immune hot phenotype IS2 and immune cold phenotype IS1 were distributed opposite to each other on the horizontal axis of the immune landscape. Furthermore, the horizontal axis of the immune landscape was negatively related to most immune cells, especially the activated dendritic cells, effector memory CD8^+^ T cells, natural killer cells, macrophages, MDSCs, and regulatory T cells, but positively correlated with Th2 cells, while the vertical axis was negatively correlated only to the memory B cells (Fig. 6B). In addition, the horizontal axis was also significantly negatively correlated with leukocyte fraction, stromal fraction, macrophage regulation, and lymphocyte infiltration, and positively correlated with proliferation, Th2 cells and resting mast cells (Additional file 1: Figure S6). The immune landscape also reflected the intra-cluster heterogeneity of ISs, particularly within IS3 and IS4. IS3 and IS4 were further divided into different subgroups based on their distribution in the immune landscape (Fig. 6C) and these subgroups also exhibited different immune cell enrichment scores. IS3A and IS4A had significantly higher scores of activated CD8^+^ T cells, activated dendritic cells, natural killer cells, macrophages, MDSCs, and regulatory T cells as compared to IS3B and IS4B (Fig. 6D, E). Thus, IS3B has a relatively cold immune status compared to IS3A, hence, could be more suitable for mRNA vaccination rather than ICI treatment. Similarly, IS4B could be a relatively promising candidate for mRNA vaccines as compared to IS4A. We divided the samples located at the extreme positions of the immune landscape into three different groups (group 1–3) and the rest of the samples were group other. Interestingly, when we performed prognostic analysis on samples in group 1–3, we found that the patients in group 3 appeared to have a relatively better prognosis than groups 1 and 2, although this trend was not significant (Fig. 6F, G). This result indicated that the distribution of patients in the immune landscape may be associated with difference in their prognosis. In general, the IS-based immune landscape can effectively reflect the status of immune components in GBM patients and may correlate with their prognosis. It is complementary to the previously defined ISs and contributes to the selection of patients for immunotherapies.

### Identification of functional immune genes modules and hub genes in GBM

**Fig. 7:**
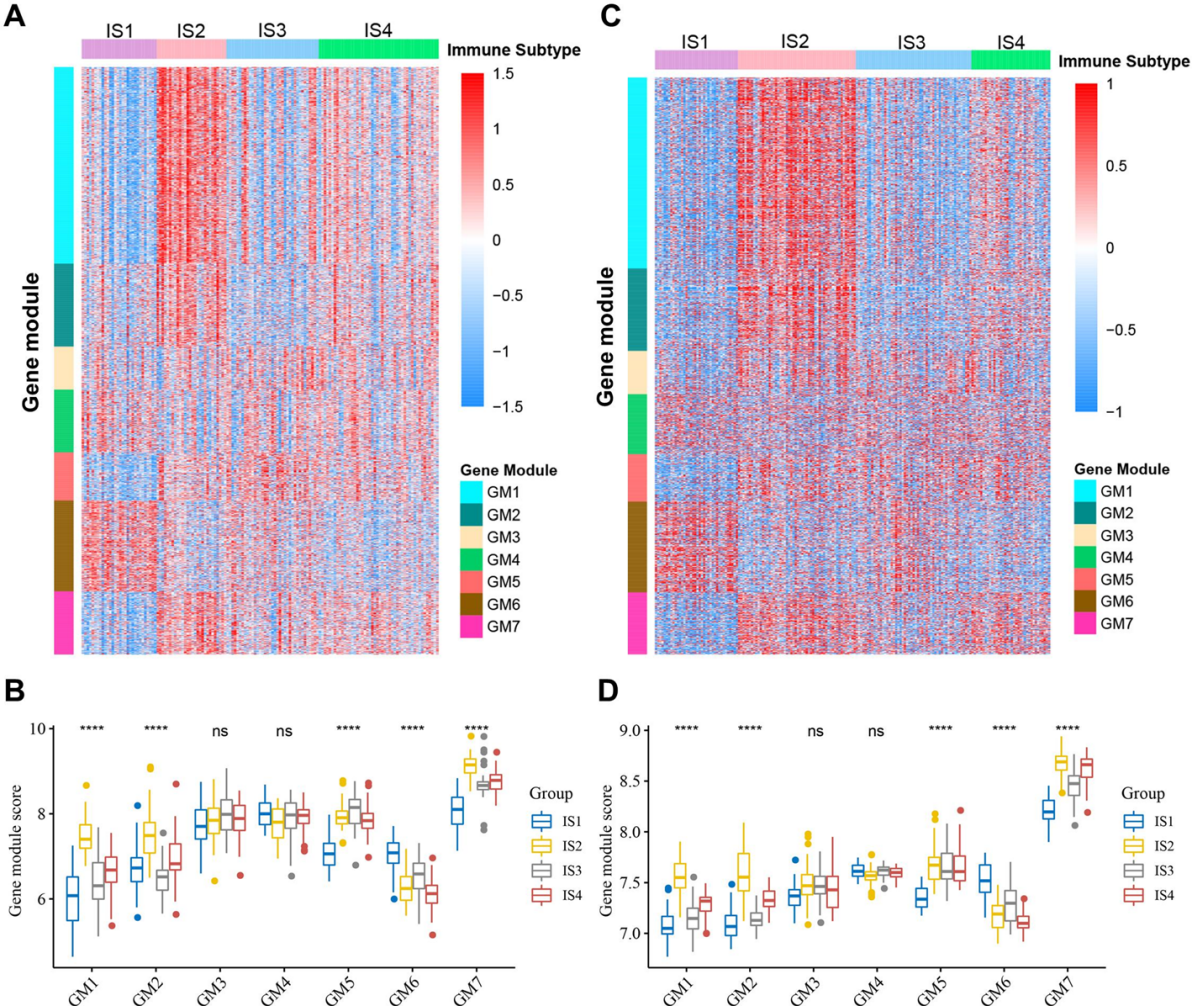
Functional immune genes modules in GBM. Heatmaps of four immune subtypes and seven gene modules in the TCGA cohort (**a**) and the REMBRANDT cohort (**c**). Genes are ordered based on the gene modules, and patients are arranged based on their immune subtypes. Box plots of the expression patterns of seven gene modules of four immune subtypes in the TCGA cohort (**b**) and the REMBRANDT cohort (**d**). ****p < 0.0001

**Fig. 8:**
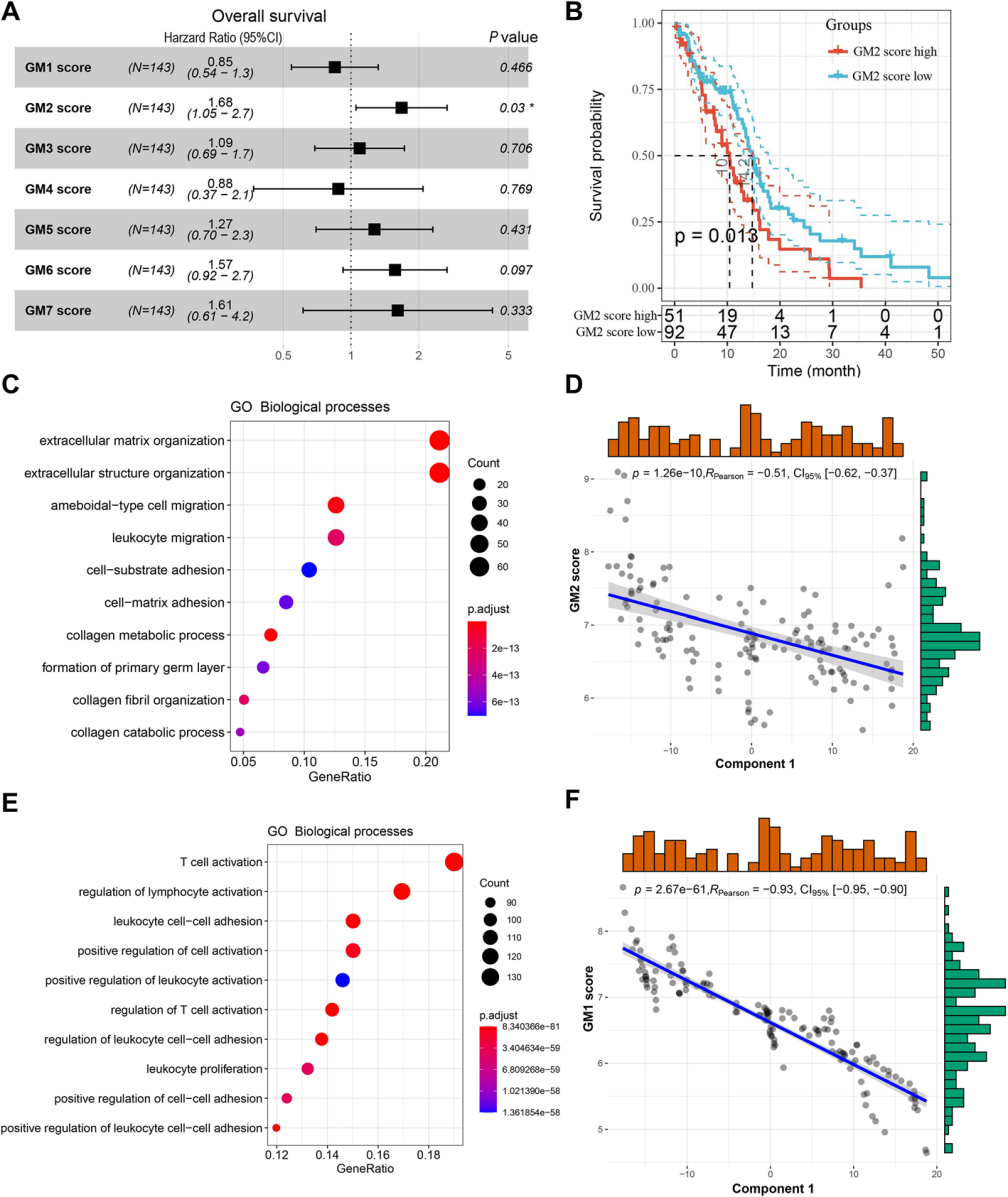
Gene module characteristics in GBM. **a** The relationship between gene modules and OS in GBM patients. **b** Kaplan–Meier curve showing OS analysis of GM2 in the TCGA cohort. GO biological process enrichment analysis of GM2 (**c**) and GM1 (**e**). Correlation between GM2 score (**d**) and GM1 score (**f**) and principal component 1 in the immune landscape

The classification of immune-related genes in GBM helps to understand the characteristics of different ISs. By performing consensus clustering of the immune-related gene expression profile in GBM, we identified seven robust GMs (GM1–GM7) (Additional file 1: Figure S7). The six previously defined ISs showed significantly different expression patterns in GMs. In the TCGA cohort, IS2 had significantly higher expression scores in GM1, GM2, and GM7, while IS1 had the opposite and also had the lowest expression score in GM5 (Fig. 7A, B). In addition, IS3 also had lower GM1 and GM2 scores, while IS4 had relatively moderate expression scores in each GM (Fig. 7A, B). The ISs in the REMBRANDT cohort also showed similar GM expression patterns (Fig. 7C, D). Subsequently, prognostic analysis showed that GM2 was significantly related to OS in GBM patients (Fig. 8A), while other GMs were not significantly correlated with OS of GBM (Additional file 1: Figure S8). Patients with high GM2 scores had significantly worse OS than patients with low GM2 scores (Fig. 8B). A similar trend was observed in the REMBRANDT cohort (Additional file 1: Figure S9). Further analysis of GO biological processes showed that the functions of GM1-GM7 correspond to T cell, reactive stroma, angiogenesis, cell morphogenesis, IFN-γ, SMAD protein phosphorylation, and antigen processing and presentation, respectively (Additional file 1: Table S5). GO enrichment analysis showed that GM2 is primarily associated with the extracellular matrix and structure (Fig. 8C). Since this GM is consistent with a previously reported 25-gene stroma signature [46], we annotated GM2 to reactive stroma. Specifically, 23 genes from the 25-gene stroma signature were included in our immune-related gene set, 18 of which were assigned to GM2. Interestingly, GM2 also showed a significant negative correlation with component 1 of the immune landscape (Fig. 8D). Furthermore, we noticed that GM1 was not only associated with T-cell activation but also with lymphocyte and leukocyte activation (Fig. 8E). GM1 likewise had a significant negative correlation with component 1 of the immune landscape (Fig. 8F). Since the activation and infiltration of T cells and other immune cells and immunosuppressive TME have important implications for the therapeutic potential of mRNA vaccines in GBM patients with specific ISs, patients with high expression of GM1 and GM2 genes may not be suitable for treatment with mRNA vaccines. To identify the hub genes in GM1 and GM2, we first constructed PPI networks for GM1 (Additional file 1: Figure S10) and GM2 genes (Additional file 1: Figure S11) separately. Subsequently, 51 hub genes were identified in GM1 (Additional file 1: Figure S12A) and the top 10 hub genes were identified in GM2 (Additional file 1: Figure S12B) using the cytoHubba plugin in the Cytoscape software. These hub genes are potential biomarkers for identifying the GBM patients that are suitable for mRNA vaccination.

### The role of immune subtypes in the GBM immunotherapy cohort

**Fig. 9:**
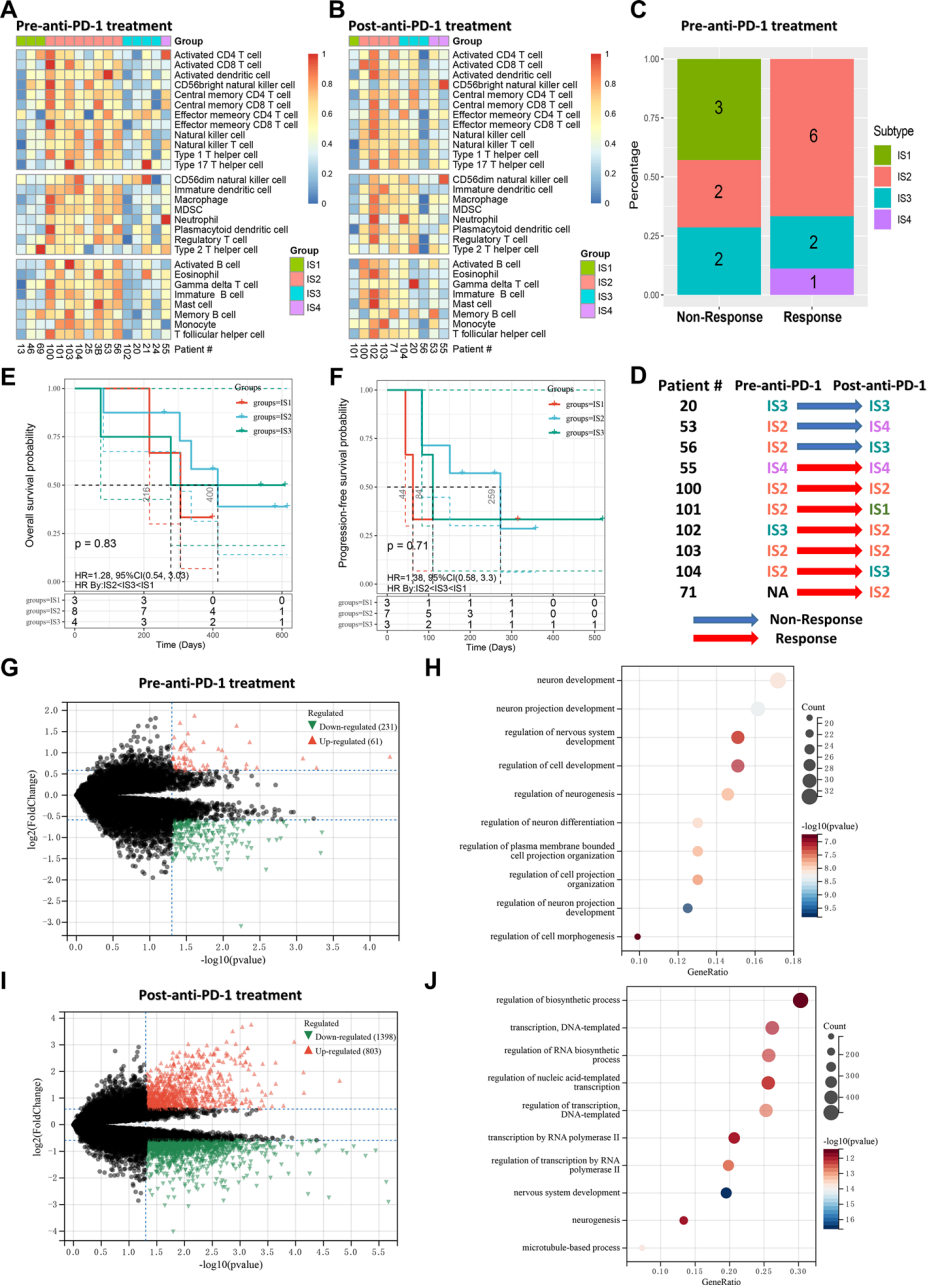
The role of immune subtypes in anti-PD-1 treatment cohort. Heatmaps of 28 previously reported immune cells infiltration among GBM immune subtypes in the pre-anti-PD-1 treatment group (**a**) and post-anti-PD-1 treatment group (**b**). **c** Distribution of immune subtypes among responders and non-responders in pre-anti-PD-1treatment samples. **d** Changes of immune subtypes in paired pre- and post-anti-PD-1 treatment samples. **e** Kaplan–Meier curve showing OS analysis of immune subtypes (IS1-IS3) in pre-anti-PD-1treatment samples. IS4 had only one sample and therefore was not included in this analysis. **f** Kaplan–Meier curve showing progression-free survival analysis of immune subtypes in pre-anti-PD-1treatment samples. IS4 had only one sample and therefore was not included in this analysis. Volcano plots showing DEGs between responders and non-responders in pre- (**g**) and post-anti-PD-1 treatment (**i**) samples. Red represented DEGs that were upregulated by responders relative to non-responders, while green represented DEGs that were downregulated. GO biological process enrichment analysis of DEGs between responders and non-responders in pre- (**h**) and post-anti-PD-1 treatment (**j**) samples

To further elucidate the potential value of IS in individualized immunotherapy, we extracted a PD-1 inhibitor treatment cohort containing 17 GBM patients from a previous study. This cohort contained 16 pre-anti-PD-1 treatment samples and 10 post-anti-PD-1 treatment samples and is the only known multi-sample GBM anti-PD-1 treatment RNA-seq cohort. Based on the PAM-based classifier established in the TCGA cohort, both pre- and post-immunotherapy samples in the PD-1 inhibitor cohort could be classified into four ISs with different immune characteristics (Fig. 9A, B). Similar to previous results, IS2 exhibited the immune hot and immunosuppressive phenotype, while the opposite was true for IS1. IS3 has a relatively cold but partially activated immune phenotype, and IS4 has a moderate TME. Analysis of the response of pre-immunotherapy samples to PD-1 inhibitors showed that all immune cold IS1 samples were non-responsive to anti-PD-1 treatment, whereas most IS2 samples were responsive and some IS3 samples were responsive (Fig. 9C). This is consistent with our previous results that IS1 and some IS3 may not be suitable for ICI treatment and that mRNA vaccines may be more promising for this group of patients. IS2 patients are ideal candidates for ICI treatment but may not be suitable for mRNA vaccines. Interestingly, after a comparative analysis of paired samples before and after anti-PD1 treatment, four IS2 cases were found to have switched to other ISs after treatment, namely TME turned from hot to cold, making two of them non-responsive to treatment (Fig. 9D). Patient #100 who turned from IS3 to IS2 responded to anti-PD-1treatment, patient #71 showed IS2 after treatment despite having no pre-treatment data, again making this patient responsive to anti-PD1 treatment (Fig. 9D). These results suggested that IS2 may have a critical impact on the response to anti-PD-1 therapy in GBM. In addition, IS2 patients appeared to have a slightly increased prognostic advantage compared to IS1 and IS3 after anti-PD-1 treatment, particularly in terms of progression-free survival time (Fig. 9E, F). Although this result was not significant due to sample size limitations, it still implied that IS may have potential prognostic significance for the prognosis of GBM immunotherapy. Using |log_2_FC|≥ 1.5 and P value < 0.05 as the threshold, 292 DEGs were identified between responders and non-responders from the pre-anti-PD-1 treatment samples (Fig. 9G, Additional file 2: Table S6), while 2201 DEGs were identified in the post-anti-PD-1 treatment samples (Fig. 9I, Additional file 2: Table S7), indicating that greater differences in gene expression emerged between responders and non-responders after anti-PD-1 treatment. KEGG and GO analysis showed that DEGs from pre-treatment samples were mainly enriched in metabolism-related pathways (Additional file 1: Figure S13A) and neuron development-related biological processes (Fig. 9H), while DEGs from post-treatment samples were mainly enriched in cell cycle-related pathways (Additional file 1: Figure S13B) and RNA biosynthesis and DNA transcription biological processes (Fig. 9J).

### Discussion

The development of mRNA vaccines relies on the identification of potential TAAs and TSAs. Huang et al. screened the potential tumor antigens for mRNA vaccine development by identifying genes that are mutated, amplified, or overexpressed in pancreatic adenocarcinoma and cholangiocarcinoma [48, 49]. However, there are currently no available candidates for mRNA vaccine development in GBM. In the present study, we used the abnormal gene expression profile and mutant gene information of GBM patients to identify nine potential mRNA vaccine antigen candidates, namely ADAMTSL4, COL6A1, CTSL, CYTH4, EGFLAM, LILRB2, MPZL2, SAA2, and LSP1. High expression of these genes was not only associated with poor prognosis of GBM but was also significantly associated with infiltration of APCs, strengthening their value as mRNA vaccine candidates. In addition, some of them have also been confirmed to have significant correlation with T-cell receptor Shannon, richness and evenness in the TCGA cohort (Additional file 1: Figure S14). Although further functional validation and preclinical evaluation are needed, the available studies support their potential as candidates. For example, a study by Zhao et al. showed that ADAMTSL4 is associated with a variety of immune-related processes, including immune cell infiltration in GBM and can be used as an independent circulating biomarker of GBM [50]. Recent studies have shown that COL6A1 expression is significantly elevated in the tumor stem cells of GBM, is involved in interaction with the extracellular matrix [51], and is a key gene associated with anti-vascular endothelial growth factor therapy [52]. CTSL is involved in ionizing radiation-induced epithelial mesenchymal transition and promotes glioma cell invasion and migration via the Akt/GSK-3 β/Snail and CUX1 pathways [53]. CYTH4 expression is significantly related to a variety of chemokines, immunomodulators, and MHC molecules [54]. In addition, high expression of EGFLAM in GBM is associated with poor prognosis, and silencing EGFLAM can inhibit proliferation, invasion, and migration of GBM cells by negatively regulating the PI3K/AKT pathway [55]. A study by Li et al. showed that LILRB2, a TME-related gene, is a potential prognostic biomarker in malignant glioma [56]. SAA2 has a unique role in promoting Th17 cell-mediated inflammatory disease [57] and plays an important role in many cancers, including GBM [57, 58]. LSP1 expression in GBM is not only closely associated with radiotherapy and chemotherapy response but is also positively correlated with immunosuppressive cell infiltration. Its high expression significantly enhances the expression of immunosuppression-related genes, including PD1, which is considered a potential new therapeutic target [59].

Given that the therapeutic response to immunotherapy including mRNA vaccine is closely related to the immune status of cancer patients, we classified GBM patients into four robust ISs based on their immune-related gene expression profile to identify those suitable for mRNA vaccines, and our ISs demonstrated good reproducibility in an independent cohort. The four ISs have different molecular and cellular characteristics, indicating completely different responses to mRNA vaccines. IS2 in both the TCGA and REMBRANDT cohorts exhibited the highest expression of ICP-related genes, indicating a strong immunosuppressive TME, which means that it could be difficult for mRNA vaccines to induce an effective immune response in IS2, but ICI therapy could be effective. In contrast, IS1 had the lowest expression of ICP-related genes in both the cohorts and elevated expression of some ICD-related genes, suggesting a more promising results of mRNA vaccination in IS1. By analyzing the infiltration of different immune cells and the overall TME in ISs, IS2 tumors were identified as hot and immunosuppressive tumors. For IS2, the use of ICIs to improve immunosuppression, thus enhancing the existing antitumor immunity seems to be a good option. Zhao et al. showed that some specific GBM patients can benefit from anti-PD-1 therapy [33], while our study confirmed that IS2 patients were more likely to respond to anti-PD-1 therapy, indicating the guiding effect of our immunophenotyping to individualized immunotherapy. Given that some current studies suggested that GBM patients have difficulty achieving survival benefits from ICI monotherapy [13, 14], the use of ICI multidrug combinations or ICI monotherapy in combination with NK cell-targeted therapy [60, 61], macrophage-targeted therapy [62], chimeric antigen receptor (CAR) T-cell therapy [63], oncolytic immunotherapy [64] or chemotherapy and radiotherapy [65] could be more effective strategies. IS1 identified as a cold phenotype, which is characterized by low immune cell infiltration and a low immunosuppressive TME, can be referred to as an immune desert phenotype. According to our study, it is difficult for IS1 patients to respond to PD-1 therapy. The use of mRNA vaccines can induce anti-tumor immune cell infiltration in IS1, thereby revitalizing the immune system of IS1 patients, and further combination with other therapies may achieve considerable clinical benefits. Therefore, IS1 patients are the ideal subjects for mRNA vaccination. IS3 has a cold TME, second only to IS1, and also has low immunosuppression, although some immune activation phenotype remains in IS3. Attempting the use of mRNA vaccines in IS3 to further activate the antitumor immune response seems promising. IS4 reflects moderate immune infiltration and moderate immunosuppressive TME. For this, mRNA vaccine combined with ICI and other treatment strategies, including CAR T-cell therapy, could be considered to activate the immune response while blocking the immunosuppressive TME. Further, graph-learning-based dimensionality reduction analysis revealed intra-cluster heterogeneity of the ISs and provided a distribution map of GBM patients, which provided complementary information on ISs and constructed an immune landscape of GBM. Both IS3 and IS4 were further divided into two subgroups in the immune landscape, with IS3B and IS4B having relatively lower immune cell infiltration and immunosuppression, thereby being more suitable for mRNA vaccination than IS3A and IS4A, while IS3A and IS4A may be more suitable for other therapies such as ICI. This complements the previously described selection of suitable patients for different immunotherapies, and combining the use of ISs and immune landscapes enables the development of better immunotherapy strategies.

Previous studies have suggested that pre-existing antitumor immunity usually improves the prognosis of cancer patients [37, 66]. However, in our study, IS2 with a hot immune phenotype had no prognostic advantage over IS1 with a cold immune phenotype. Given the strong immunosuppression in IS2, determining the relative advantage between immunostimulatory and immunosuppressive factors in GBM patients is critical for evaluating the prognosis. Immunosuppressive TME has a critical impact on the prognosis of GBM patients [67–69], consistent with this, we identified GM2 (reactive stroma module) as a risk factor contributing to the poor prognosis of GBM patients. In addition, group 3, which is at the extreme end of the immune landscape, has a relatively good prognosis and low infiltration of the stimulatory and suppressive immune cells. Therefore, immunosuppressive factors seem to have a critical impact on the prognosis of patients with GBM. Considering the overall immunosuppressive TME of GBM [67–69], the combination of mRNA vaccines with ICI is a recommended strategy even in IS1 with a cold immunophenotype.

In the recently established pan-cancer immunophenotyping, Thorsson et al. defined six immune classes applicable to 33 different types of tumors, according to which, most of the GBM cases were classified as C4 [42]. Clearly, our study has shown that the immune status of different GBM patients is very different. Therefore, a simple classification using pan-cancer immunophenotyping may not be adequate for a highly heterogeneous tumor like GBM. Our ISs provide immunophenotyping specifically for GBM and are complementary to the previously defined pan-cancer immunophenotyping. Moreover, the GM1 and GM2 gene scores were significantly negatively correlated with component 1 of the immune landscape, implying that high expression of GM1 and GM2 genes is associated with high level of immune cell infiltration as well as immunosuppression. To further enhance clinical applicability, we identified hub genes in GM1 and GM2. Patients with high expression of these genes may show poor response to mRNA vaccines.

Notably, in the recent 2021 WHO classification, only IDH wildtype GBM is considered as GBM, while the previously known IDH-mutant GBM is classified as IDH-mutant high-grade astrocytoma [70]. Therefore, we analyzed IDH-wildtype GBM again after excluding 6 cases of IDH-mutant GBM in the TCGA cohort based on this classification. The results of the analysis of IDH-wildtype GBM were found to be highly consistent with the results described previously (Additional file 1: Figures S15–S19). In addition, there are some limitations in this study. First, we were unable to determine whether the identified tumor antigens were expressed by GBM cells or other immune/stromal cells. Second, we could not validate the response of different ISs to GBM vaccine because we didn’t find RNA-Seq data from a large clinical trial of GBM vaccine. Although the tumor antigens and ISs identified in this study require further experimental validation, this study provides important information for the development and clinical application of mRNA vaccine and individualized immunotherapy for GBM in the future.

## Conclusions

In conclusion, we identified ADAMTSL4, COL6A1, CTSL, CYTH4, EGFLAM, LILRB2, MPZL2, SAA2, and LSP1 as candidate tumor antigens for mRNA vaccine development for GBM and established four robust GBM immune subtypes to identify the potential subjects for different immunotherapies. This study provides a theoretical framework for future GBM mRNA vaccine development, selection of patients to be vaccinated, and better individualized immunotherapeutic strategy designs.

## Supplementary Information

**Additional** **file** **1:** **Figure** **S1.** Identification of tumor antigens associated with GBM prognosis. Kaplan-Meier curves comparing RFS for groups with different expression of ADAMTSL4 (**a**), COL6A1 (**b**), CTSL (**c**), CYTH4 (**d**), EGFLAM (**e**), LILRB2 (**f**), MPZL2 (**g**), SAA2 (**h**), and LSP1 (**i**) in GBM. Red lines represented high gene expression, blue represented low gene expression. **Figure** **S2.** Identification of tumor antigens associated with infiltration of antigen-presenting cells in TCGA cohort. The correlation between the expression levels of ADAMTSL4 (**a**), COL6A1 (**b**), CTSL (**c**), CYTH4 (**d**), EGFLAM (**e**), LILRB2 (**f**), MPZL2 (**g**), SAA2 (**h**), and LSP1 (**i**) and infiltration levels of dendritic cells and macrophages in GBM. **Figure** **S3.** A two gene signature based on LSP1 and ADAMTSL4, risk score = LSP1*0.33421+ ADAMTSL4*0.12244. **a** Risk distribution, survival status and LSP1 and ADAMTSL4 expression in GBM patients. **b** Kaplan-Meier curve comparing OS of different risk in GBM patients. **c** ROC curve showing good predictive performance. **Figure** **S4.** Correlation between immune subtypes and prognosis of GBM. **a** Kaplan-Meier curve comparing OS of different immune subtypes in the TCGA cohort. **b** Kaplan-Meier curve comparing OS of different immune subtypes in the REMBRANDT cohort. **Figure** **S5.** Association of immune subtypes with TMB and mutation in GBM. TMB (**a**) and mutation number (**b**) of different immune subtypes in GBM. **c** The top 10 frequently mutated genes in GBM immune subtypes. **Figure** **S6.** Correlation between principal component 1/2 and 21 immune-related molecular signatures. **Figure** **S7.** Identification of functional immune genes modules in GBM. Cumulative distribution function curve (**a**), delta area curve (**b**), and consensus heatmap (**c**) of immune-related gene expression profile in the TCGA cohort. **Figure** **S8.** Relationship between GMs and prognosis of GBM patients in the TCGA cohort. Kaplan-Meier curves showing OS analysis of GM1 (**a**), GM3 (**b**), GM4 (**c**), GM5 (**d**), GM6 (**e**) and GM7 (**f**) in the TCGA cohort. Red lines represented high GM scores, blue represented low GM scores. **Figure** **S9.** Kaplan-Meier curve showing OS analysis of GM2 in the REMBRANDT cohort. **Figure** **S10.** Protein-protein interaction network for GM1 genes. **Figure** **S11.** Protein-protein interaction network for GM2 genes. **Figure** **S12.** Immune hub genes in GBM. **a** 51 hub genes in GM1. **b** The top 10 hub genes in GM2. **Figure** **S13.** KEGG pathway analysis of DEGs between responders and non-responders. **a** KEGG pathway analysis of DEGs in pre-anti-PD-1 treatment samples. **b** KEGG pathway analysis of DEGs in post-anti-PD-1 treatment samples. **Figure** **S14.** Seven candidate antigens detected in the TCGA cohort in relation to T cell receptors Shannon, richness and evenness. **Figure** **S15.** Association of immune subtypes with TMB and mutation in IDH-wildtype GBM. TMB (**a**) and mutation number (**b**) of different immune subtypes in IDH-wildtype GBM. **Figure** **S16.** Association between immune subtypes and immunomodulators in IDH-wildtype GBM. **a** Differences in expression levels of ICP-related genes among immune subtypes in IDH-wildtype GBM. **b** Differences in expression levels of ICD-related genes among immune subtypes in IDH-wildtype GBM. *p < 0.05, **p < 0.01, ***p < 0.001, ****p < 0.0001. **Figure** **S17.** Cellular and molecular characteristic of immune subtypes in IDH-wildtype GBM. **a** Heatmap of 28 previously reported immune cell signatures scores among immune subtypes in IDH-wildtype GBM. **b** Differences of 28 immune cell signatures scores among immune subtypes in IDH-wildtype GBM. **c** The distribution of **I**DH-wildtype GBM four immune subtypes in the pan-cancer immune subtypes. **d** 21 immune-related molecular signatures with significant differences among IDH-wildtype GBM immune subtypes. *p < 0.05, **p < 0.01, ***p < 0.001, ****p < 0.0001. **Figure** **S18.** The immune landscape of IDH-wildtype GBM. **a** The immune landscape of IDH-wildtype GBM. Each dot represents a patient, and different colors represent different immune subtypes. The horizontal axis represents the principal component 1, and the vertical axis represents the principal component 2. **b** Correlation between principal component 1/2 and 28 immune cell enrichment scores. **c** Immune landscape of the subgroups of IDH-wildtype GBM immune subtypes. Differences of 28 immune cells enrichment scores in the subgroups of IS3 (**d**) and IS4 (**e**). Immune landscape of samples from three extreme locations (**f**) and their prognostic status (**g**). ^-^*p *≥ 0.1, ^·^*p* < 0.1, *p < 0.05, **p < 0.01, ***p < 0.001. **Figure** **S19.** Functional immune genes modules in IDH-wildtype GBM. **a** Heatmap of four immune subtypes and seven gene modules in IDH-wildtype GBM. Genes are ordered based on the gene modules, and patients are arranged based on their immune subtypes. **b** Box plots of the expression patterns of seven gene modules of four immune subtypes in IDH-wildtype GBM. ****p < 0.0001. **Table** **S1.** Clinical characteristics of GBM patients in the TCGA cohort, REMBRANDT cohort and PD-1 inhibitor cohort. **Table** **S4.** IGP was estimated for each immune subtype in the validation cohort. **Table** **S5.** Functional enrichment analysis of gene modules.

**Additional** **file** **2: Table S2**. The clinical information of TCGA cohort. **Table** **S3.** Immune-related genes. **Table** **S6.** Differentially expressed genes between responders and non-responders in pre-anti-PD-1 treatment samples. **Table** **S7.** Differentially expressed genes between responders and non-responders in post-anti-PD-1 treatment samples.

## Data Availability

All data generated and described in this article are available from the corresponding web servers, and are freely available to any scientist wishing to use them for noncommercial purposes, without breaching participant confidentiality. Further information is available from the corresponding author on reasonable request.

## Abbreviations

GBM: Glioblastoma
OS: Overall survival
RFS: Relapse-free survival
ICI: Immune checkpoint inhibitor
TAA: Tumor-associated antigens
TSA: Tumor-specific antigens
mRNA: Messenger RNA
APC: Antigen-presenting cell
IS: Immune subtype
TCGA: The Cancer Genome Atlas
PAM: Partition around medoids
IGP: Intra-group proportion
GM: Gene module
ssGSEA: Single-sample gene set enrichment analysis
PP: Protein–protein interaction
ADAMTSL4: ADAMTS-like 4
COL6A1: Collagen type VI alpha 1 chain
CTSL: Cathepsin L
CYTH4: Cytohesin 4
EGFLAM: EGF like, fibronectin type III and laminin G domains
LILRB2: Leukocyte immunoglobulin-like receptor B2
MPZL2: Myelin protein zero like 2
SAA2: Serum amyloid A2
LSP1: Lymphocyte specific protein 1
ICP: Immune checkpoint
ICD: Immunogenic cell death
TME: Tumor microenvironment
MDSCs: Myeloid-derived suppressor cells
DEGs: Differentially expressed genes
KEGG: Kyoto encyclopedia of genes and genomes
GO: Gene ontology
