# Supplementary material for: Tumor antigens and immune subtypes of glioblastoma: the fundamentals of mRNA vaccine and individualized immunotherapy development
Source: J Big Data. 2022 Jul 14;9(1):92. doi: 10.1186/s40537-022-00643-x (PMC9281265; doi:10.1186/s40537-022-00643-x)
Supplement: Supplementary file 1 — Additional file 1: Figure S1. Identification of tumor antigens associated with GBM prognosis. Kaplan-Meier curves comparing RFS for groups with different expression of ADAMTSL4 (a), COL6A1 (b), CTSL (c), CYTH4 (d), EGFLAM (e), LILRB2 (f), MPZL2 (g), SAA2 (h), and LSP1 (i) in GBM. Red lines represented high gene expression, blue represented low gene expression. Figure S2. Identification of tumor antigens associated with infiltration of antigen-presenting cells in TCGA cohort. The correlation between the expression levels of ADAMTSL4 (a), COL6A1 (b), CTSL (c), CYTH4 (d), EGFLAM (e), LILRB2 (f), MPZL2 (g), SAA2 (h), and LSP1 (i) and infiltration levels of dendritic cells and macrophages in GBM. Figure S3. A two gene signature based on LSP1 and ADAMTSL4, risk score = LSP1*0.33421+ ADAMTSL4*0.12244. a Risk distribution, survival status and LSP1 and ADAMTSL4 expression in GBM patients. b Kaplan-Meier curve comparing OS of different risk in GBM patients. c ROC curve showing good predictive performance. Figure S4. Correlation between immune subtypes and prognosis of GBM. a Kaplan-Meier curve comparing OS of different immune subtypes in the TCGA cohort. b Kaplan-Meier curve comparing OS of different immune subtypes in the REMBRANDT cohort. Figure S5. Association of immune subtypes with TMB and mutation in GBM. TMB (a) and mutation number (b) of different immune subtypes in GBM. c The top 10 frequently mutated genes in GBM immune subtypes. Figure S6. Correlation between principal component 1/2 and 21 immune-related molecular signatures. Figure S7. Identification of functional immune genes modules in GBM. Cumulative distribution function curve (a), delta area curve (b), and consensus heatmap (c) of immune-related gene expression profile in the TCGA cohort. Figure S8. Relationship between GMs and prognosis of GBM patients in the TCGA cohort. Kaplan-Meier curves showing OS analysis of GM1 (a), GM3 (b), GM4 (c), GM5 (d), GM6 (e) and GM7 (f) in the TCGA cohort. Red lines represent [file 40537_2022_643_MOESM1_ESM.docx]

**Supplementary figures and tables**


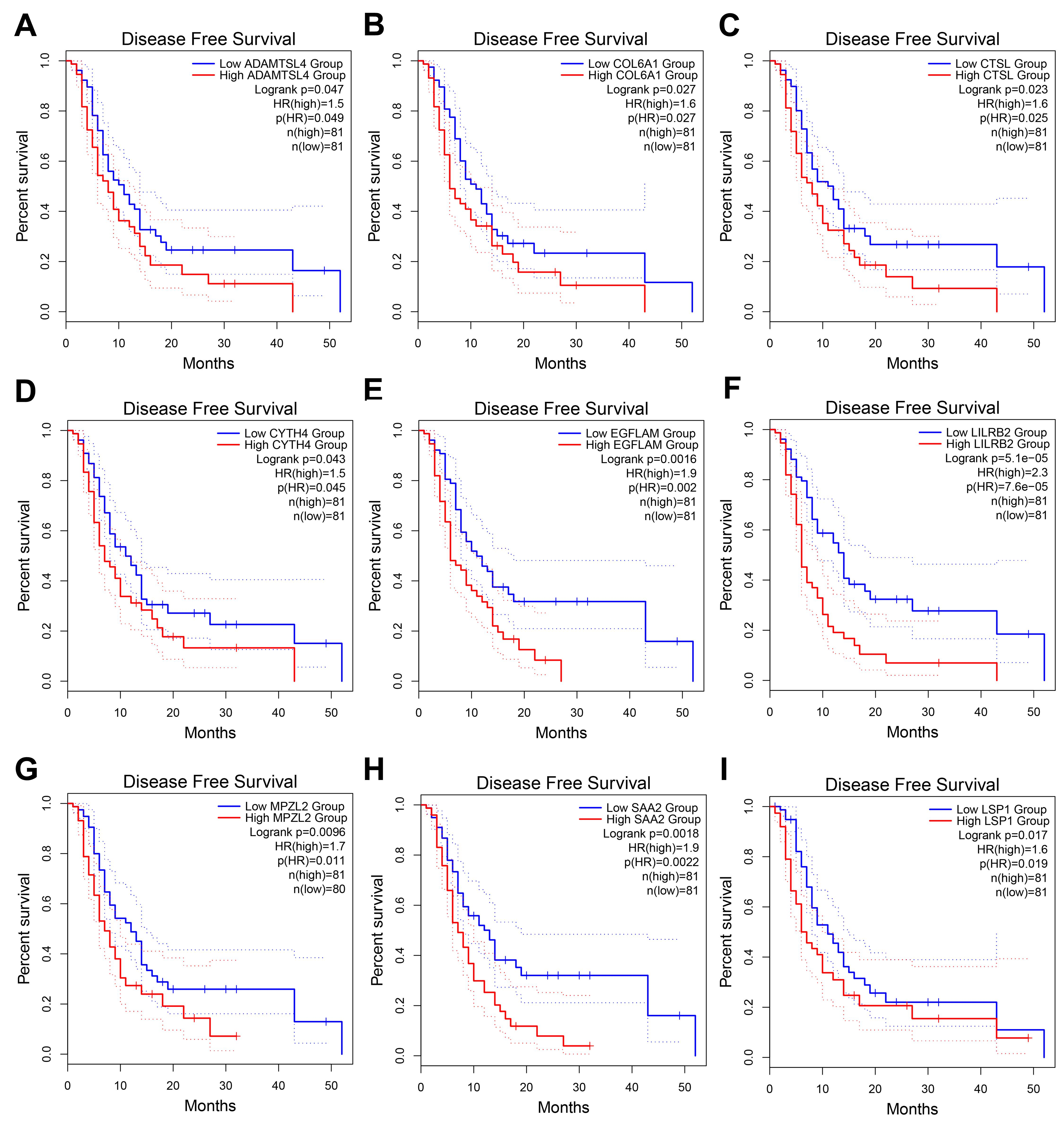


**Figure S1. Identification of tumor antigens associated with GBM prognosis. a-i** Kaplan-Meier curves comparing RFS for groups with different expression of ADAMTSL4 (**a**), COL6A1 (**b**), CTSL (**c**), CYTH4 (**d**), EGFLAM (**e**), LILRB2 (**f**), MPZL2 (**g**), SAA2 (**h**), and LSP1 (**i**) in GBM. Red lines represented high gene expression, blue represented low gene expression.


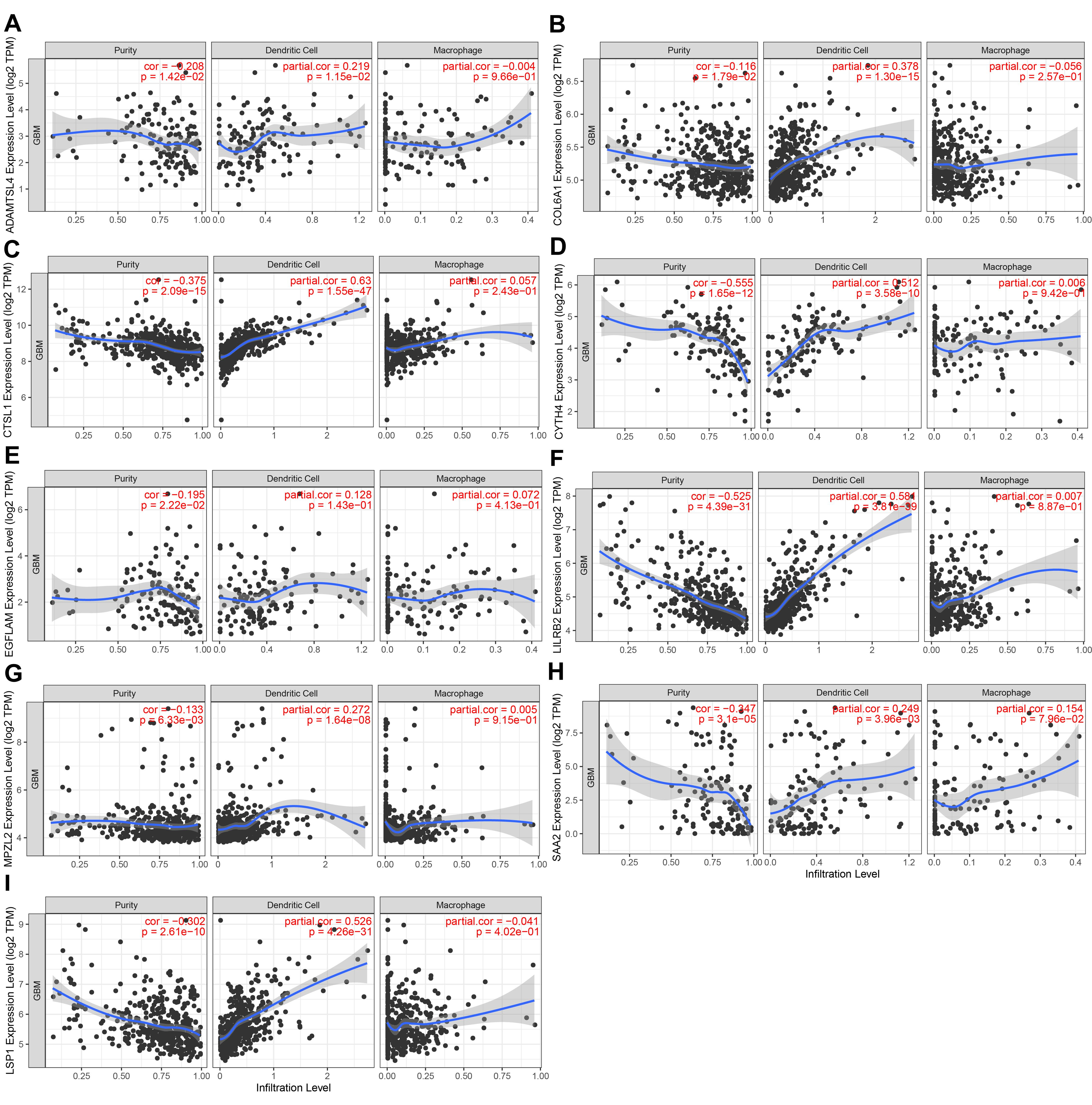


**Figure S2. Identification of tumor antigens associated with infiltration of antigen-presenting cells in TCGA cohort. a-i** The correlation between the expression levels of ADAMTSL4 (**a**), COL6A1 (**b**), CTSL (**c**), CYTH4 (**d**), EGFLAM (**e**), LILRB2 (**f**), MPZL2 (**g**), SAA2 (**h**), and LSP1 (**i**) and infiltration levels of dendritic cells and macrophages in GBM.


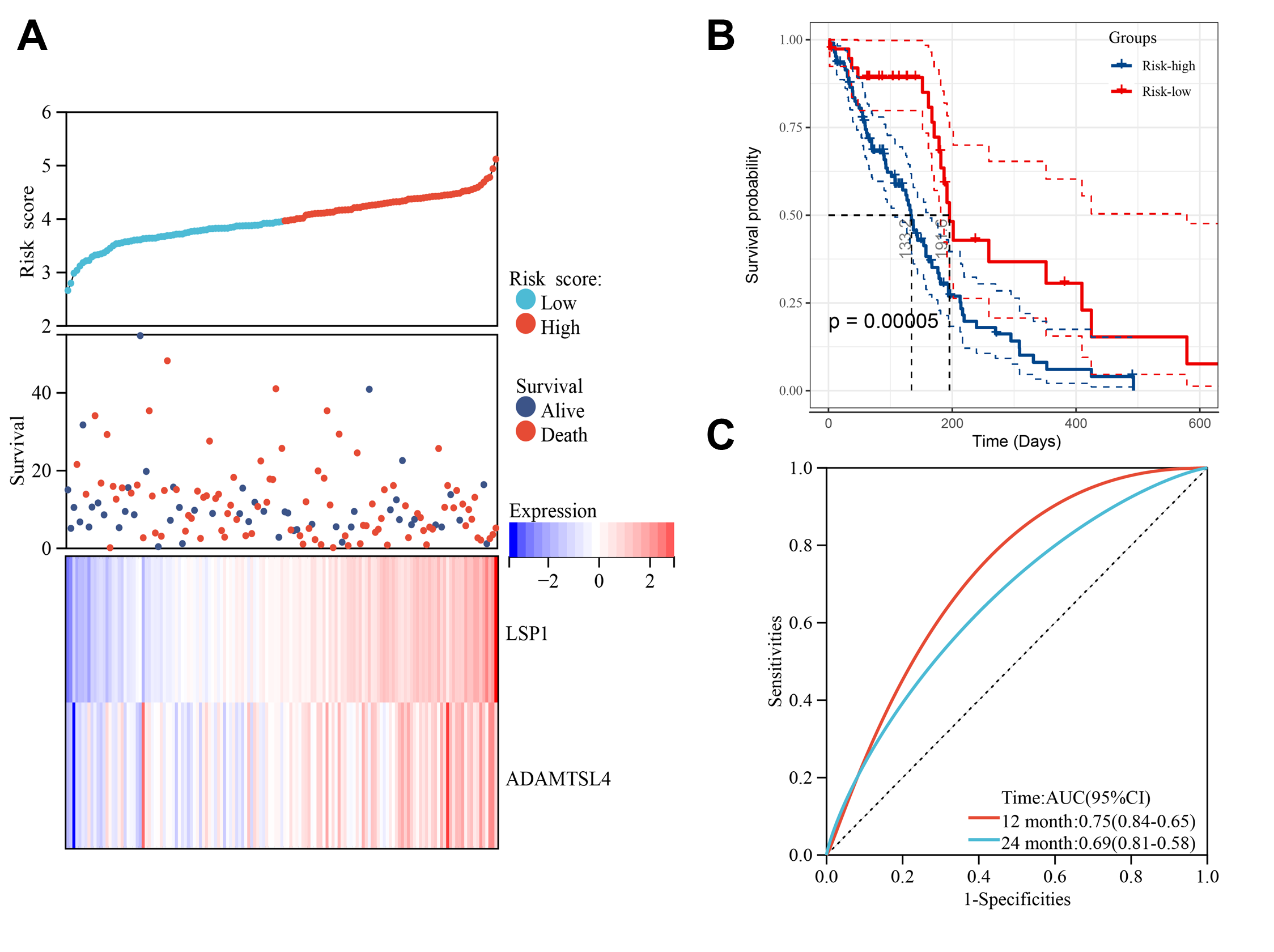


**Figure S3. A two gene signature based on LSP1 and ADAMTSL4, risk score = LSP1*0.33421+ ADAMTSL4*0.12244. a** Risk distribution, survival status and LSP1 and ADAMTSL4 expression in GBM patients. **b** Kaplan-Meier curve comparing OS of different risk in GBM patients. **c** ROC curve showing good predictive performance.


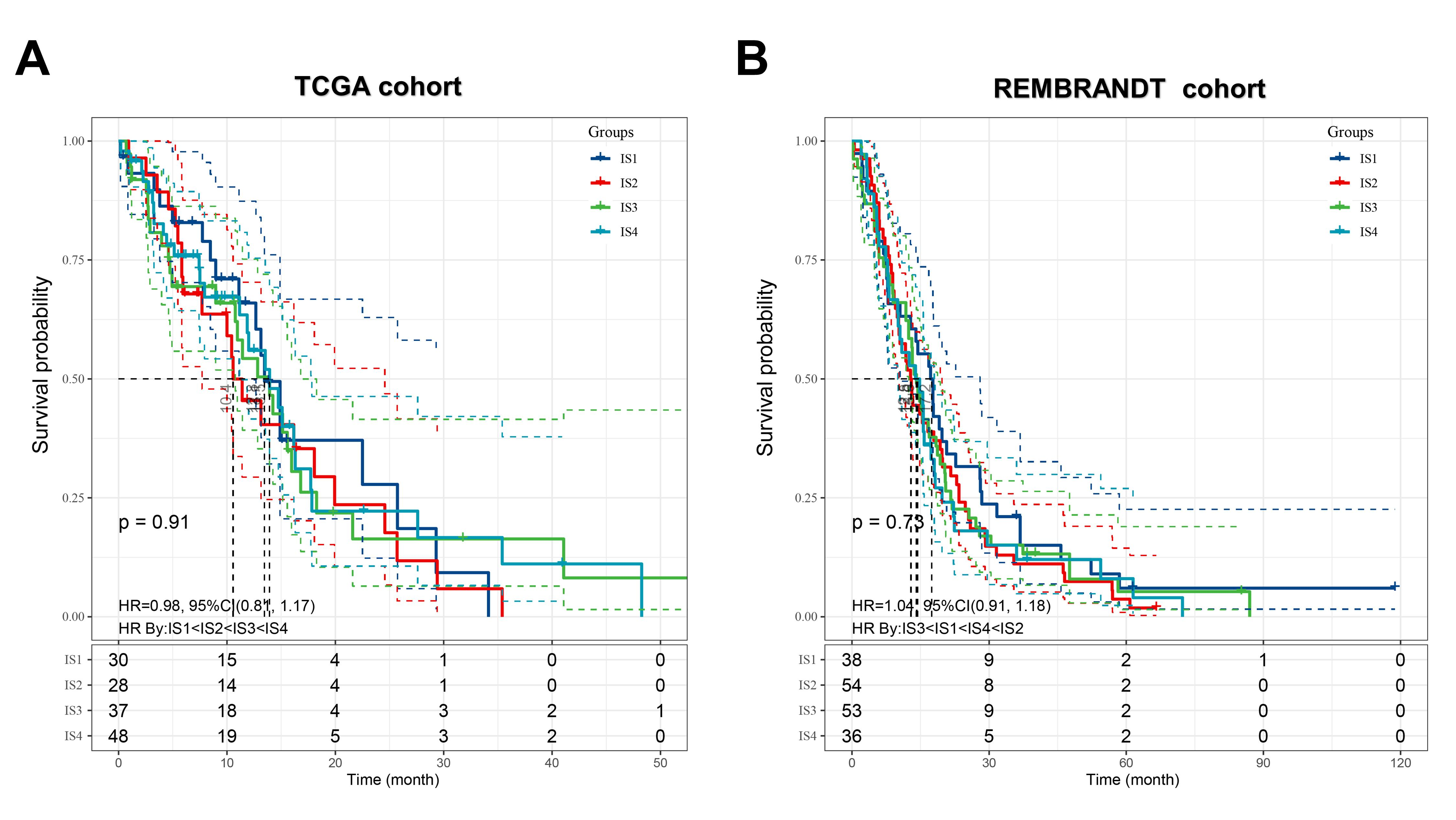


**Figure S4.** **Correlation between immune subtypes and prognosis of GBM. a** Kaplan-Meier curve comparing OS of different immune subtypes in the TCGA cohort. **b** Kaplan-Meier curve comparing OS of different immune subtypes in the REMBRANDT cohort.


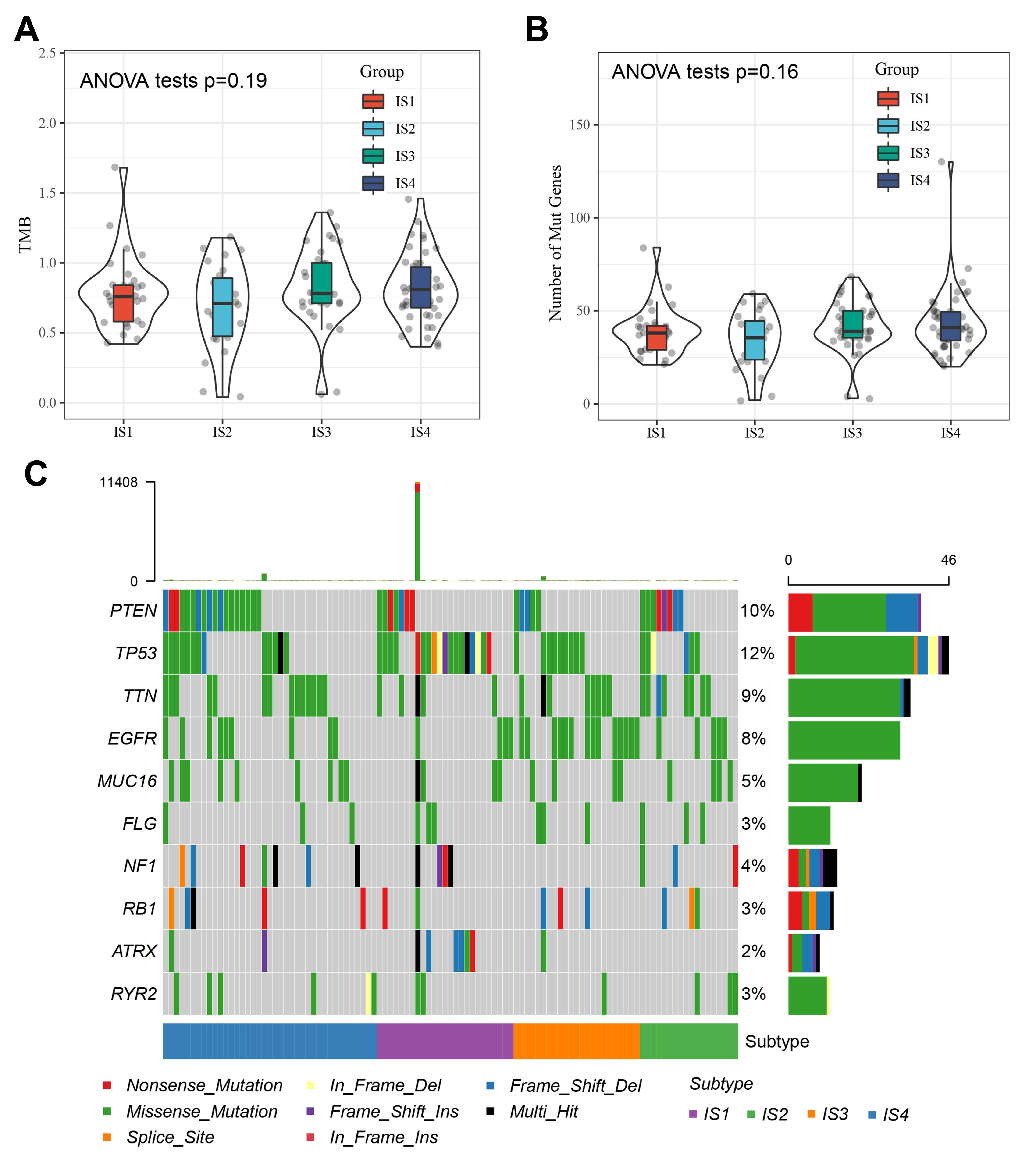


**Figure S5. Association of immune subtypes with TMB and mutation in GBM. a,b** TMB (**a**) and mutation number (**b**) of different immune subtypes in GBM. **c** The top 10 frequently mutated genes in GBM immune subtypes.


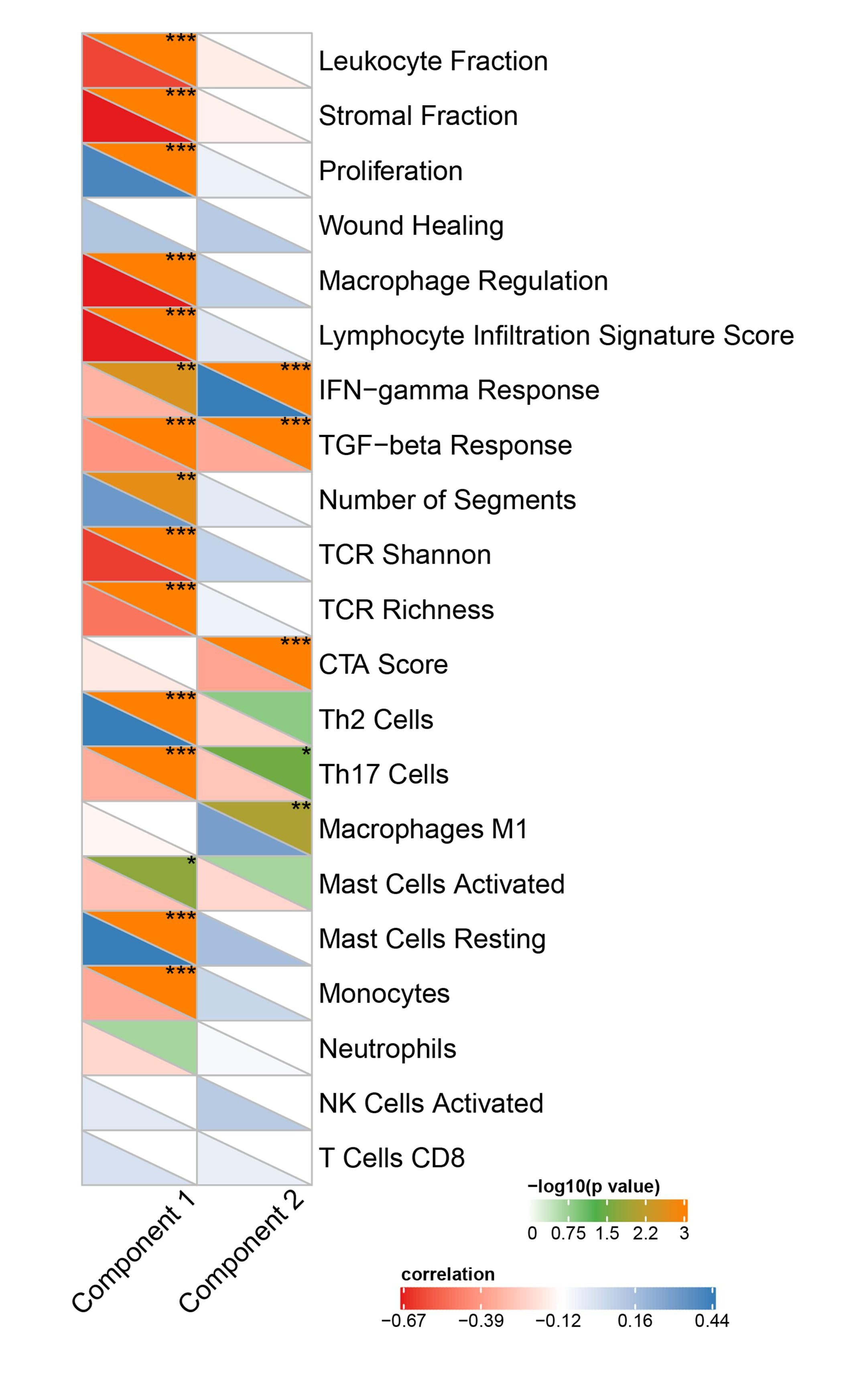


**Figure S6. Correlation between principal component 1/2 and 21 immune-related molecular signatures.**


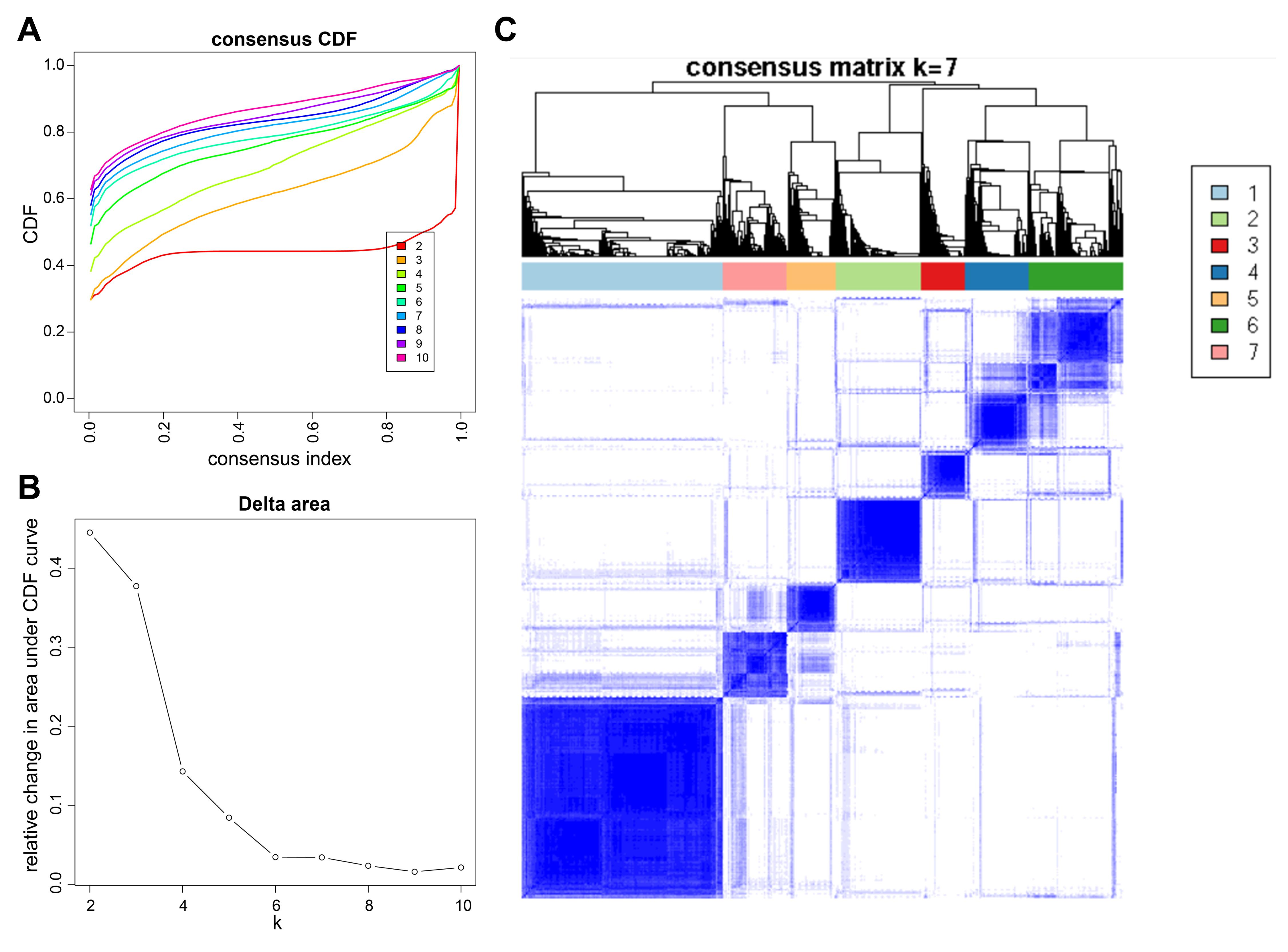


**Figure S7. Identification of functional immune genes modules in GBM.** **a-c** Cumulative distribution function curve (**a**), delta area curve (**b**), and consensus heatmap (**c**) of immune-related gene expression profile in the TCGA cohort.


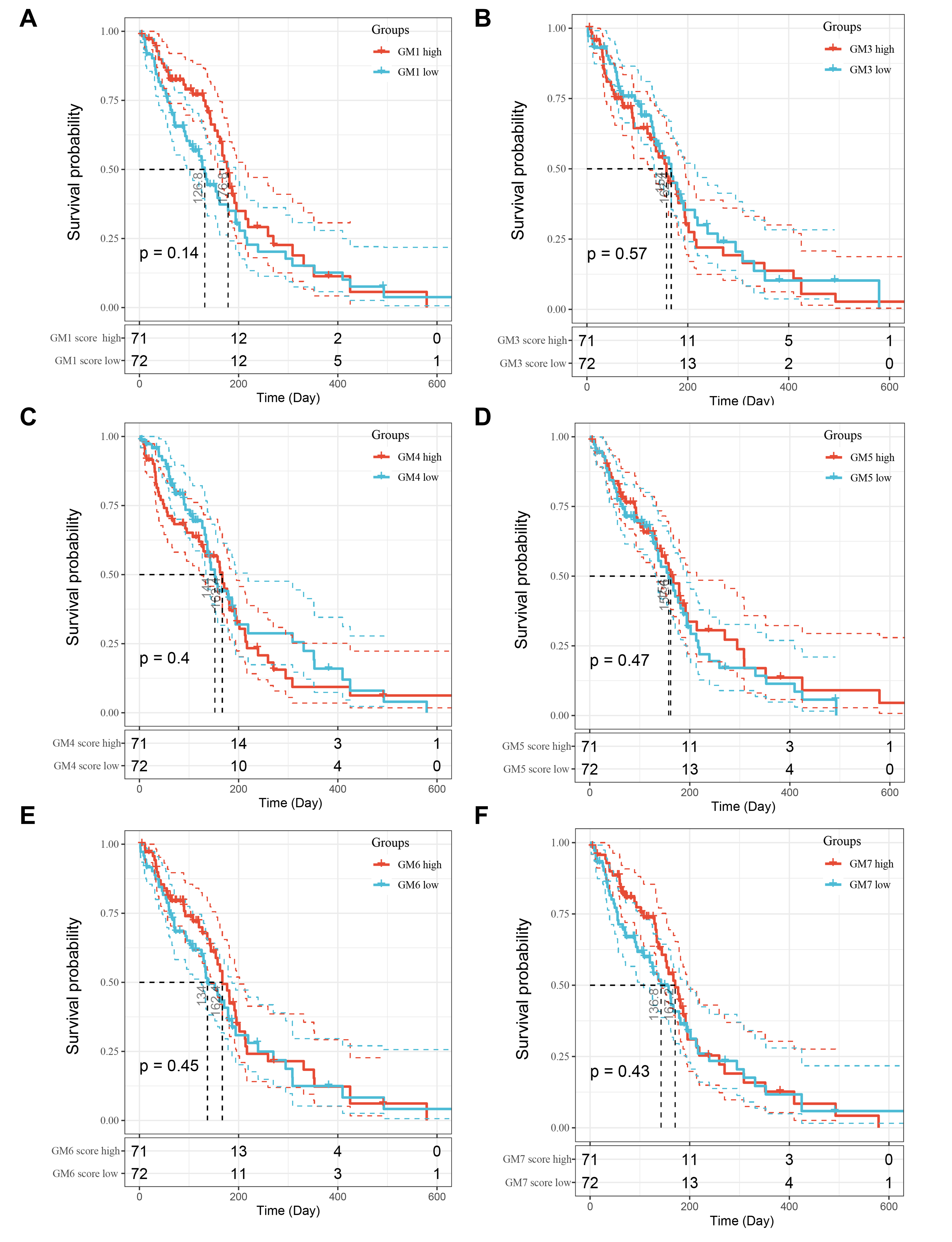


**Figure S8. Relationship between GMs and prognosis of GBM patients in the TCGA cohort. a-f** Kaplan-Meier curves showing OS analysis of GM1 (**a**), GM3 (**b**), GM4 (**c**), GM5 (**d**), GM6 (**e**) and GM7 (**f**) in the TCGA cohort. Red lines represented high GM scores, blue represented low GM scores.


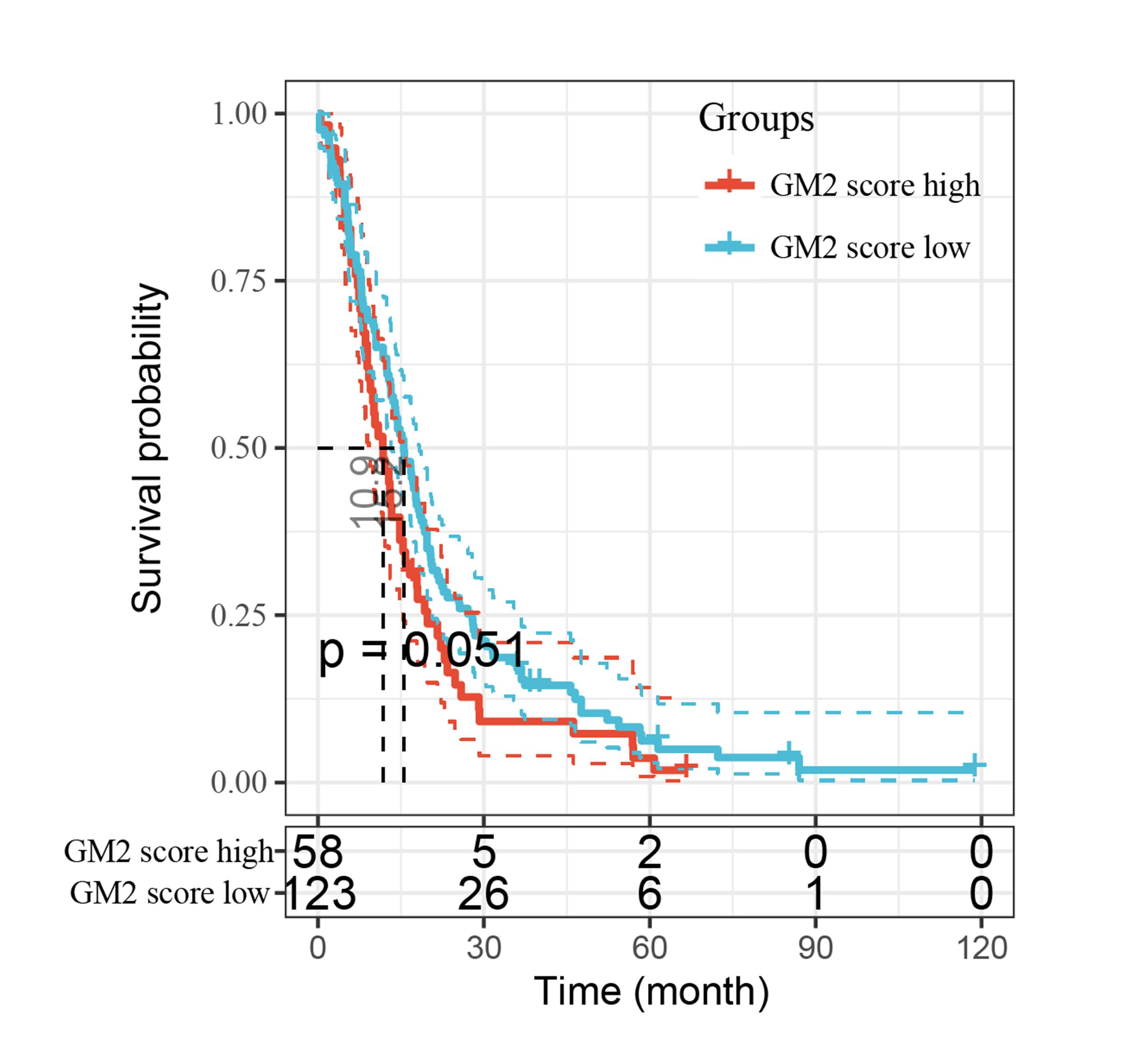


**Figure S9. Kaplan-Meier curve showing OS analysis of GM2 in the REMBRANDT cohort.**


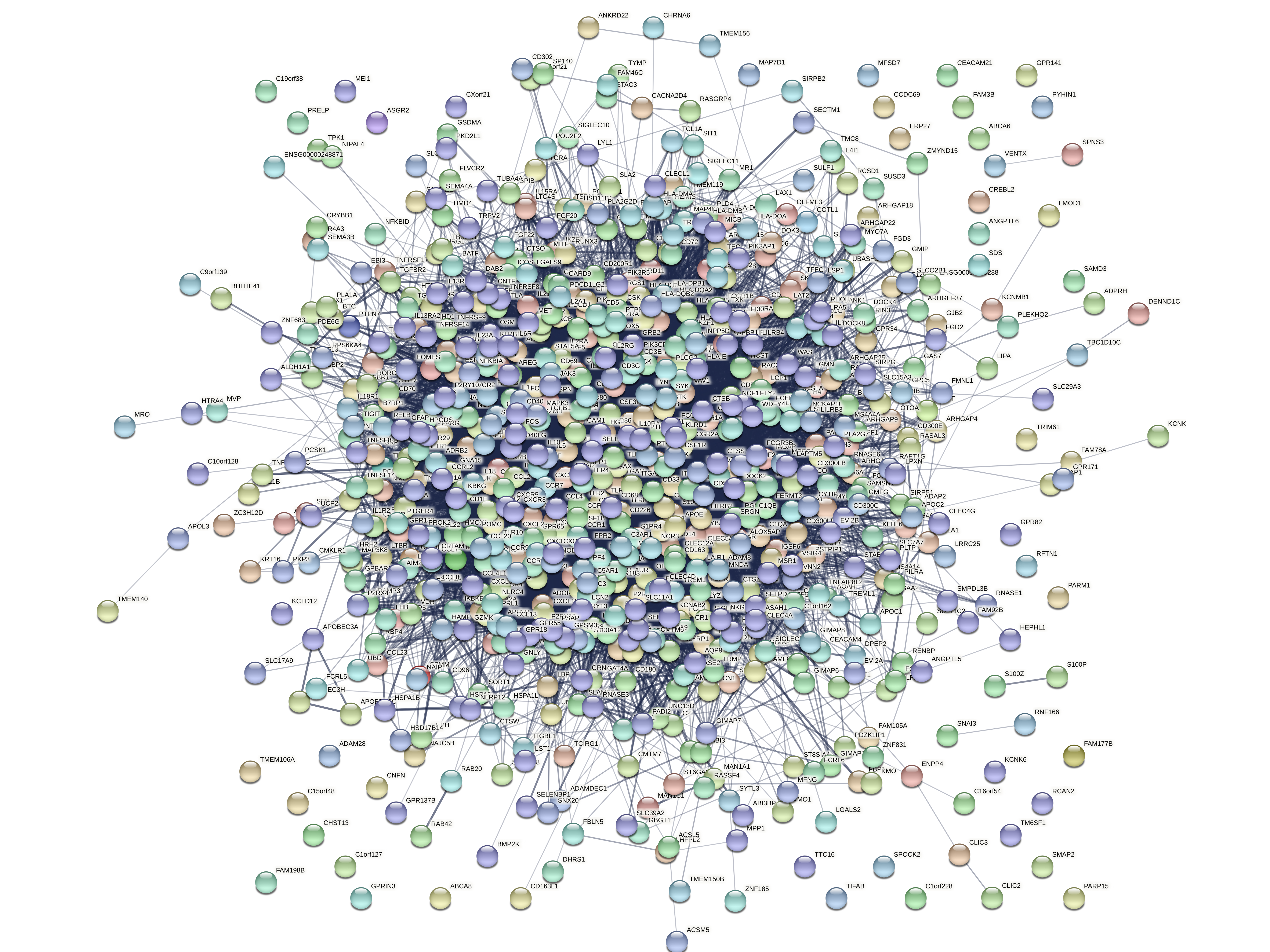


**Figure S10.** **Protein-protein interaction network for GM1 genes.**


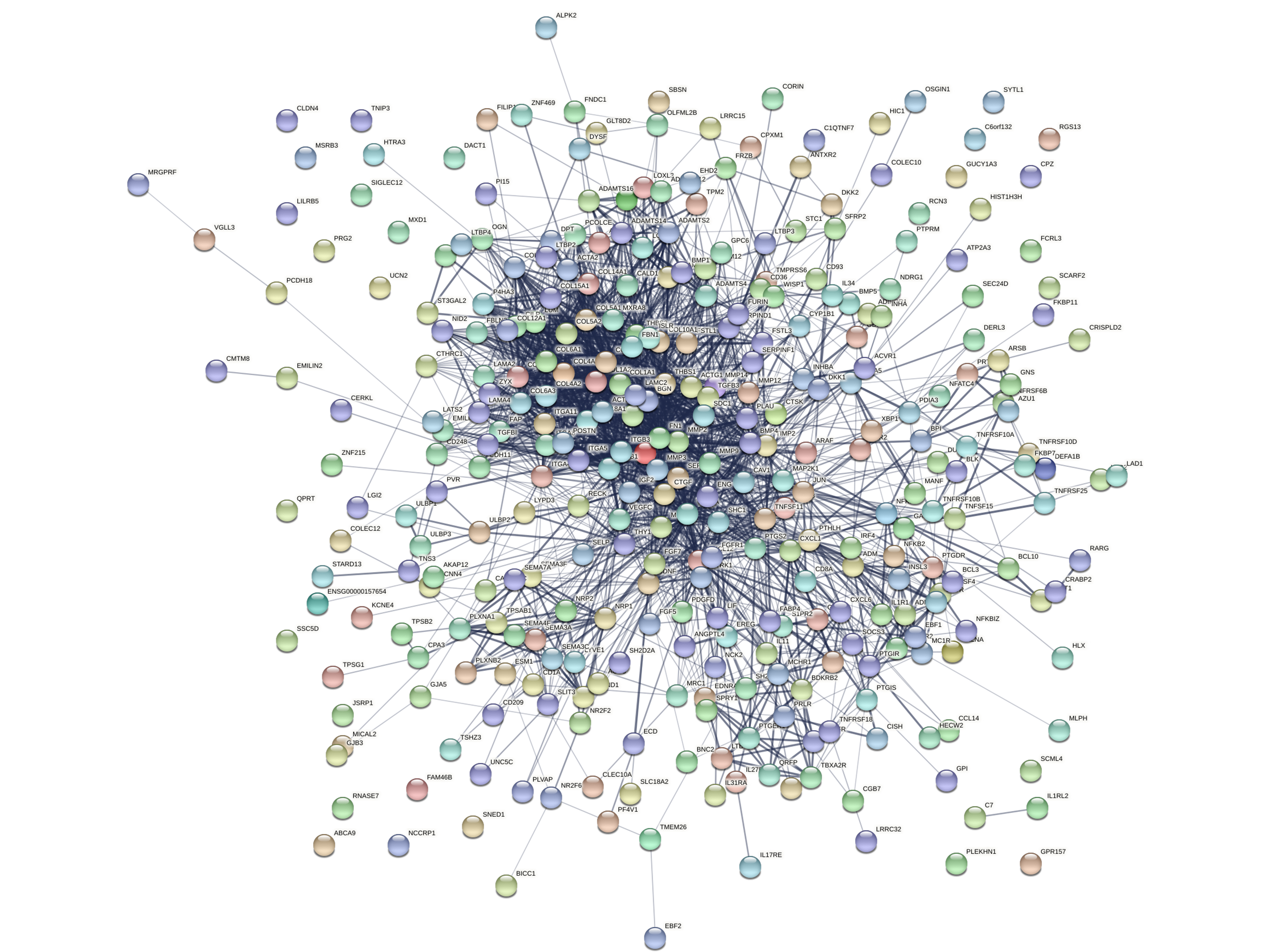


**Figure S11.** **Protein-protein interaction network for GM2 genes.**


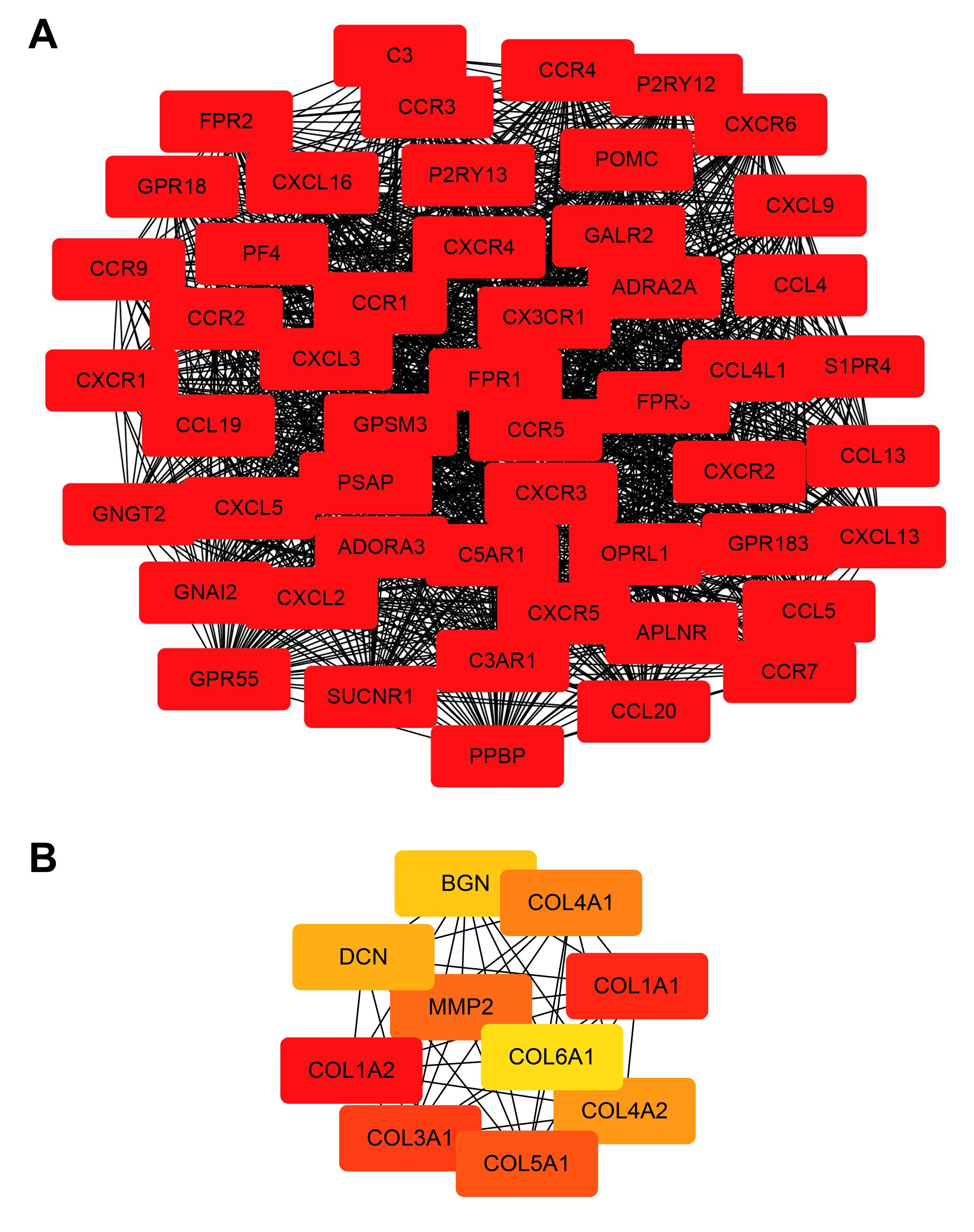


**Figure S12. Immune hub genes in GBM. a** 51 hub genes in GM1. **b** The top 10 hub genes in GM2.


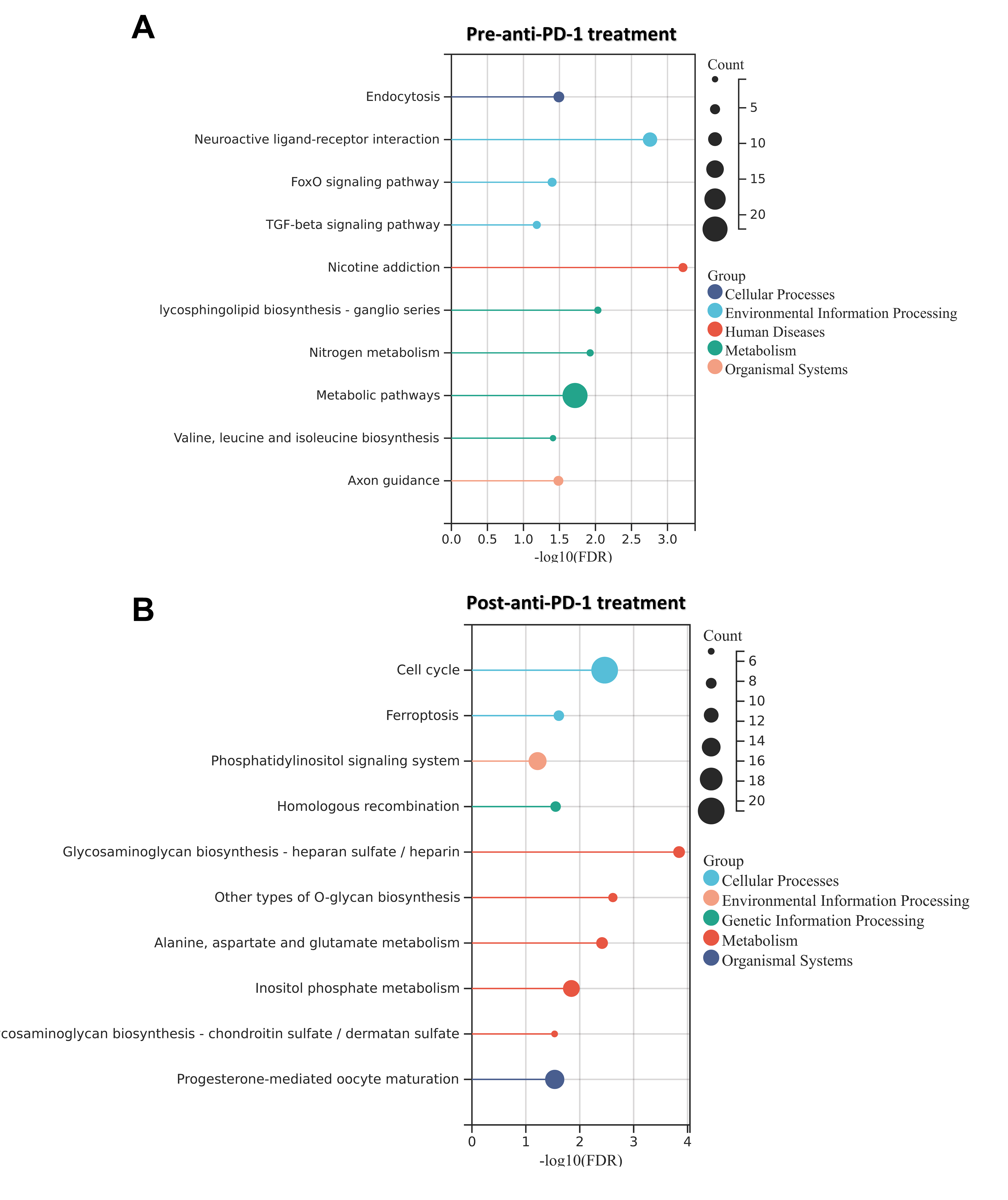


**Figure S13. KEGG pathway analysis of DEGs between responders and non-responders. a** KEGG pathway analysis of DEGs in pre-anti-PD-1 treatment samples. **b** KEGG pathway analysis of DEGs in post-anti-PD-1 treatment samples.


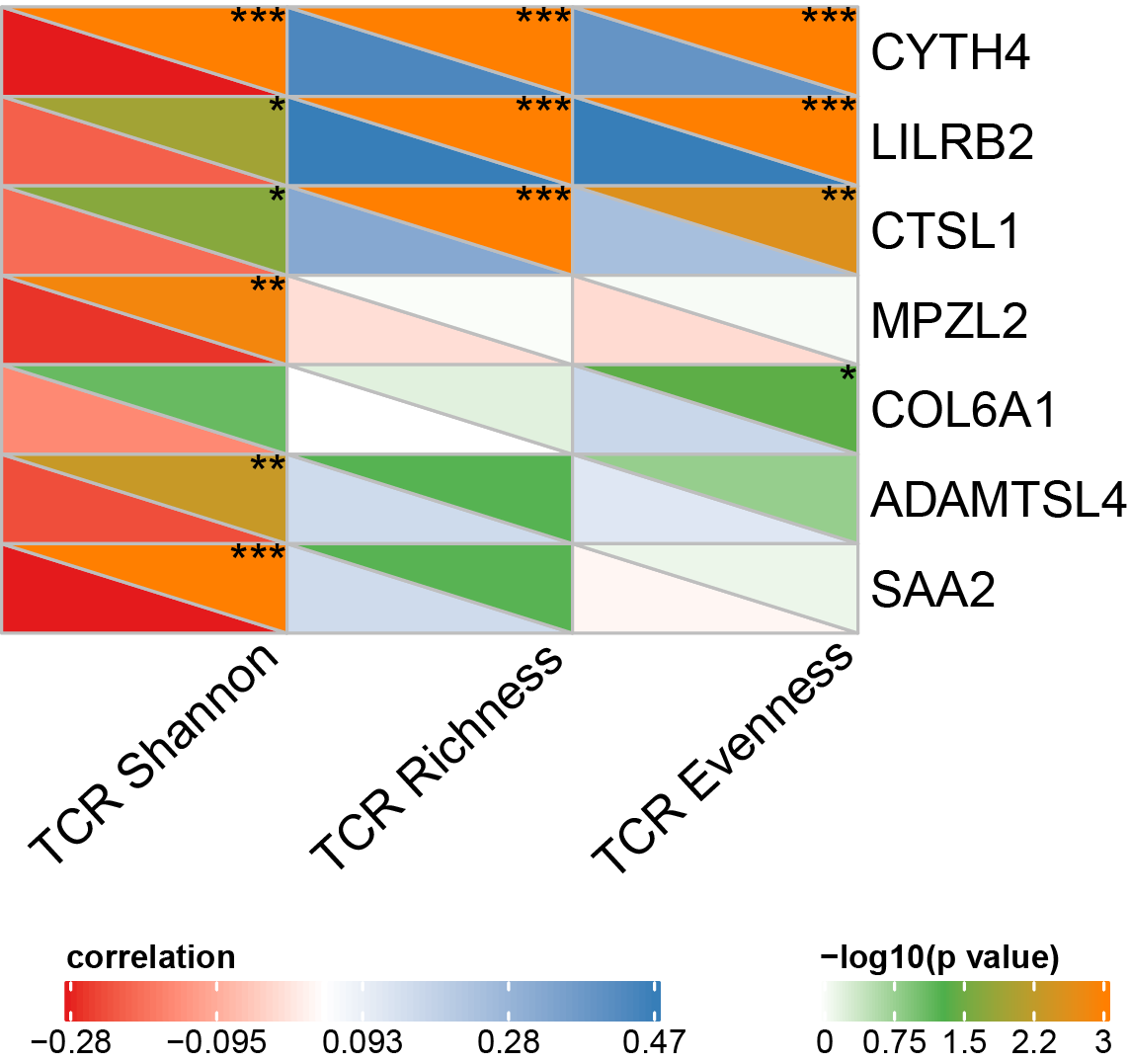


**Figure S14. Seven candidate antigens detected in the TCGA cohort in relation to T cell receptors Shannon, richness and evenness.**


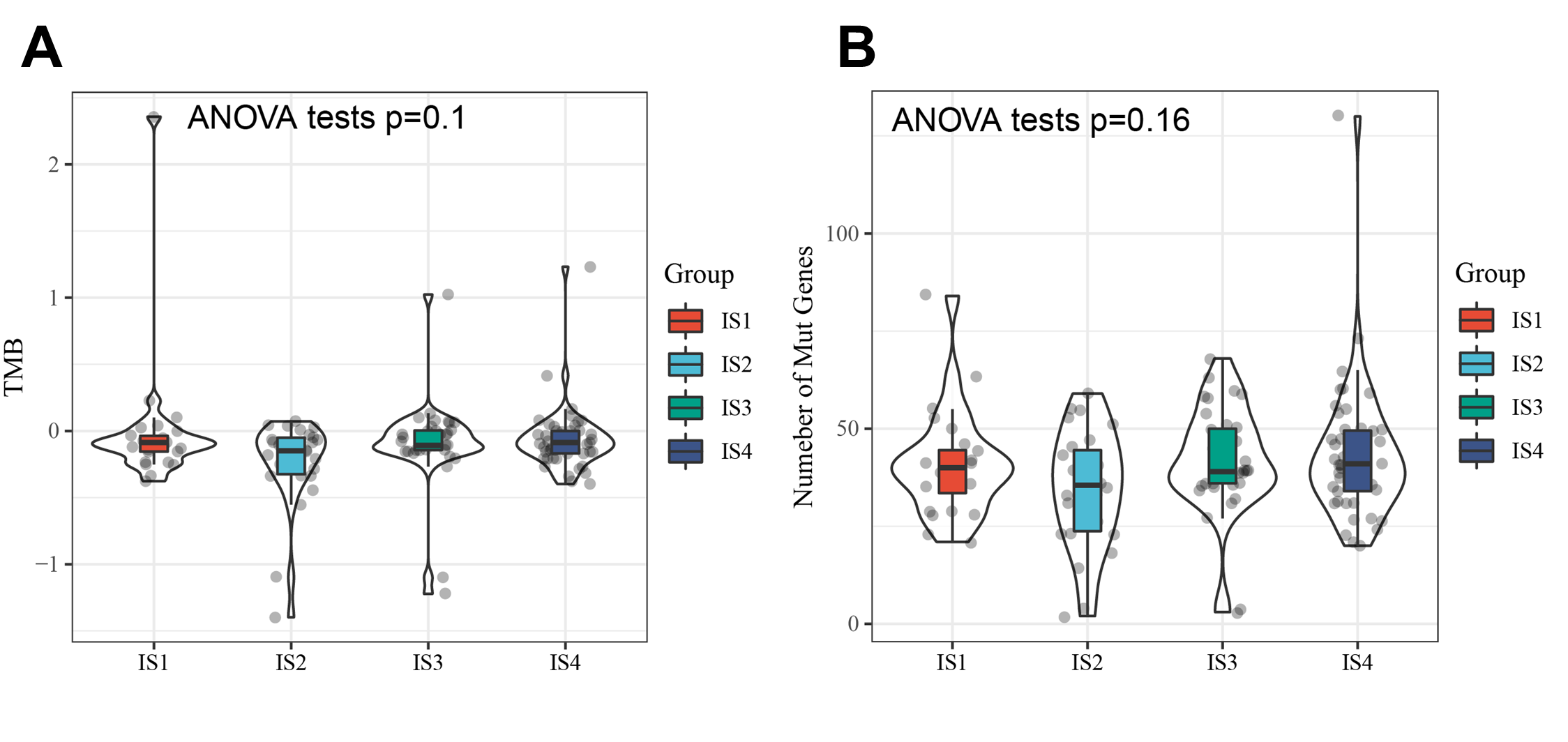


**Figure S15. Association of immune subtypes with TMB and mutation in IDH-wildtype GBM. a,b** TMB (**a**) and mutation number (**b**) of different immune subtypes in IDH-wildtype GBM.


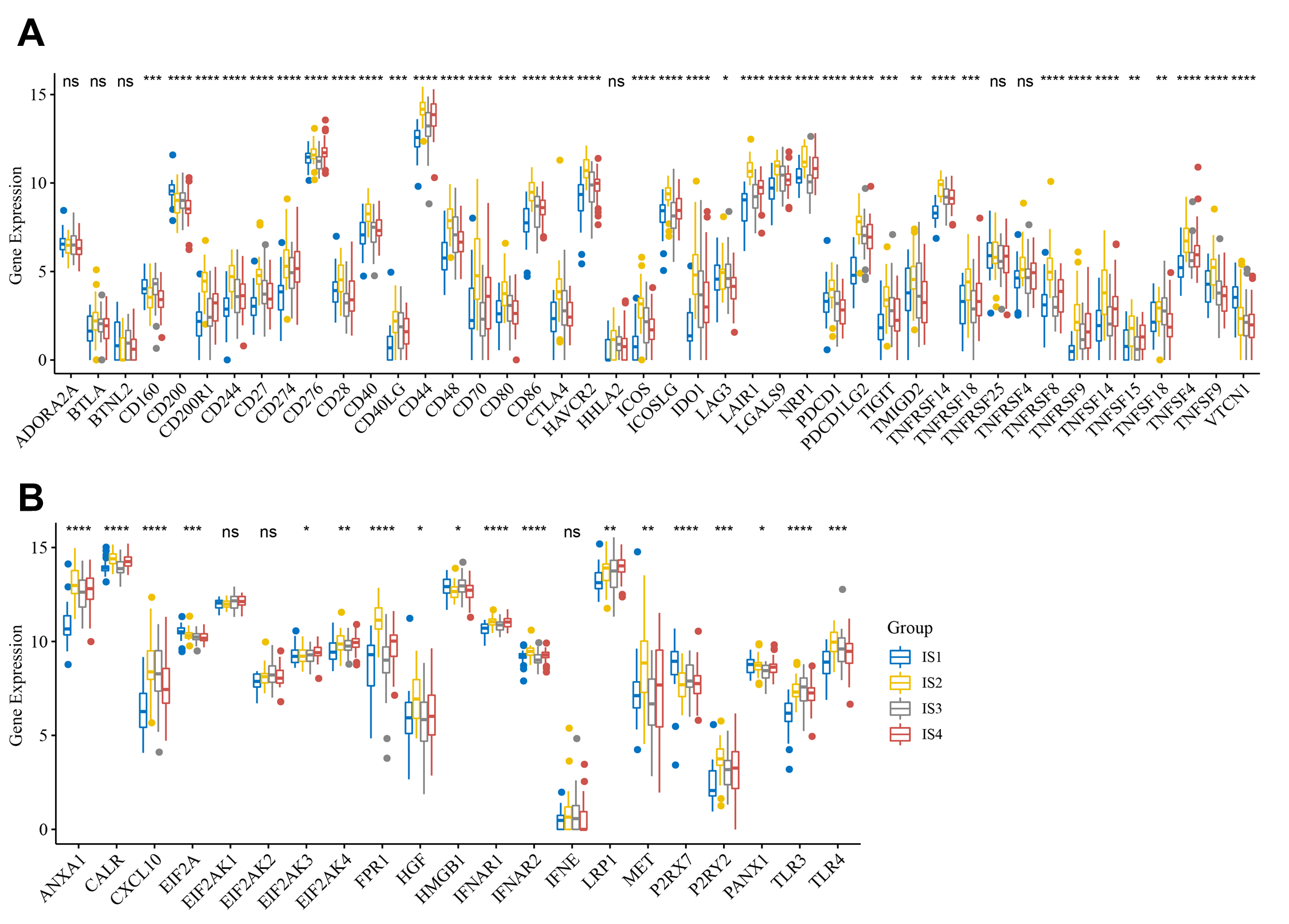


**Figure S16. Association between immune subtypes and immunomodulators in IDH-wildtype GBM. a** Differences in expression levels of ICP-related genes among immune subtypes in IDH-wildtype GBM. **b** Differences in expression levels of ICD-related genes among immune subtypes in IDH-wildtype GBM. * *p* < 0.05, ** *p* < 0.01, *** *p* < 0.001, **** *p* < 0.0001


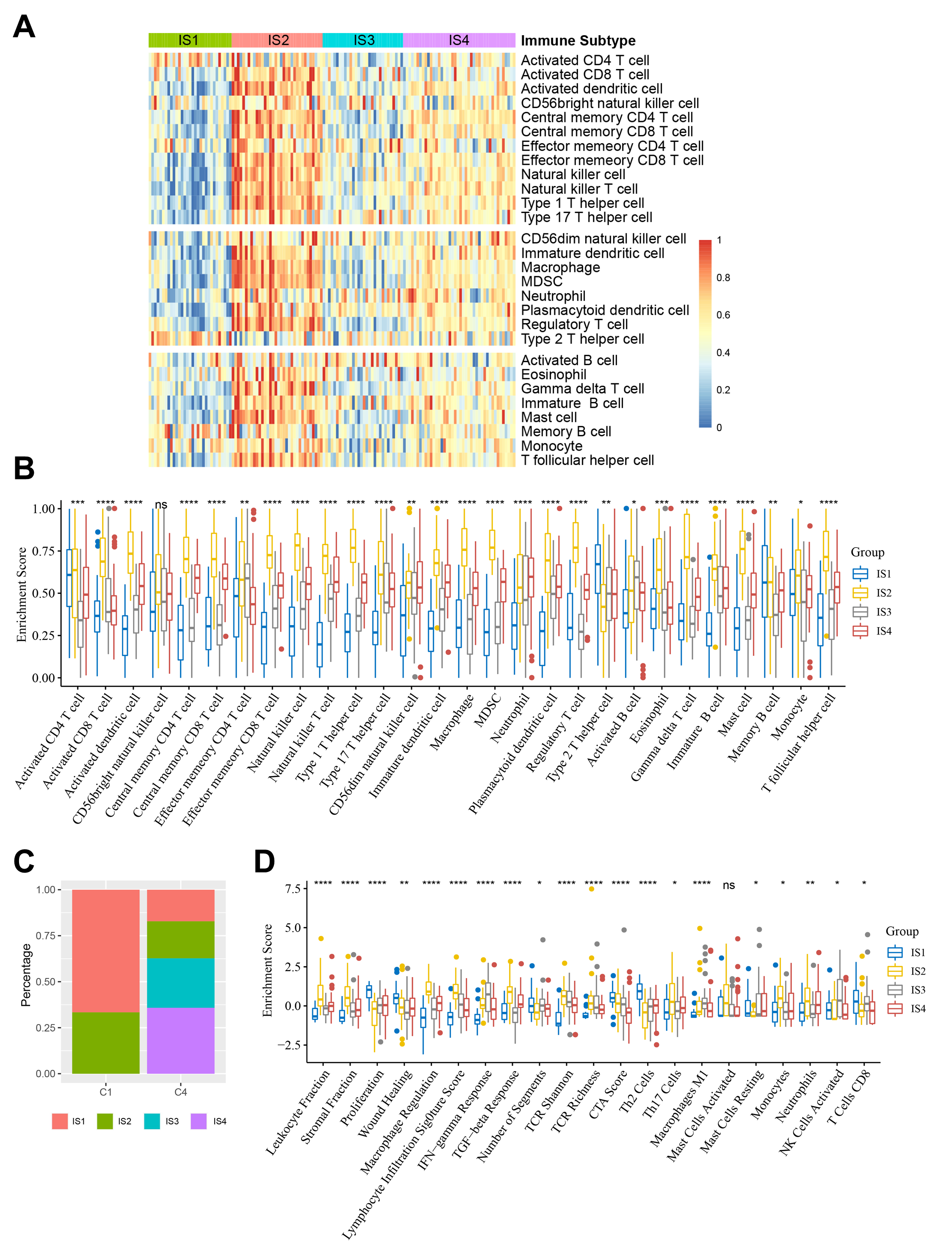


**Figure S17. Cellular and molecular characteristic of immune subtypes in IDH-wildtype GBM. a** Heatmap of 28 previously reported immune cell signatures scores among immune subtypes in IDH-wildtype GBM. **b** Differences of 28 immune cell signatures scores among immune subtypes in IDH-wildtype GBM. **c** The distribution of **I**DH-wildtype GBM four immune subtypes in the pan-cancer immune subtypes. **d** 21 immune-related molecular signatures with significant differences among IDH-wildtype GBM immune subtypes. * *p* < 0.05, ** *p* < 0.01, *** *p* < 0.001, **** *p* < 0.0001


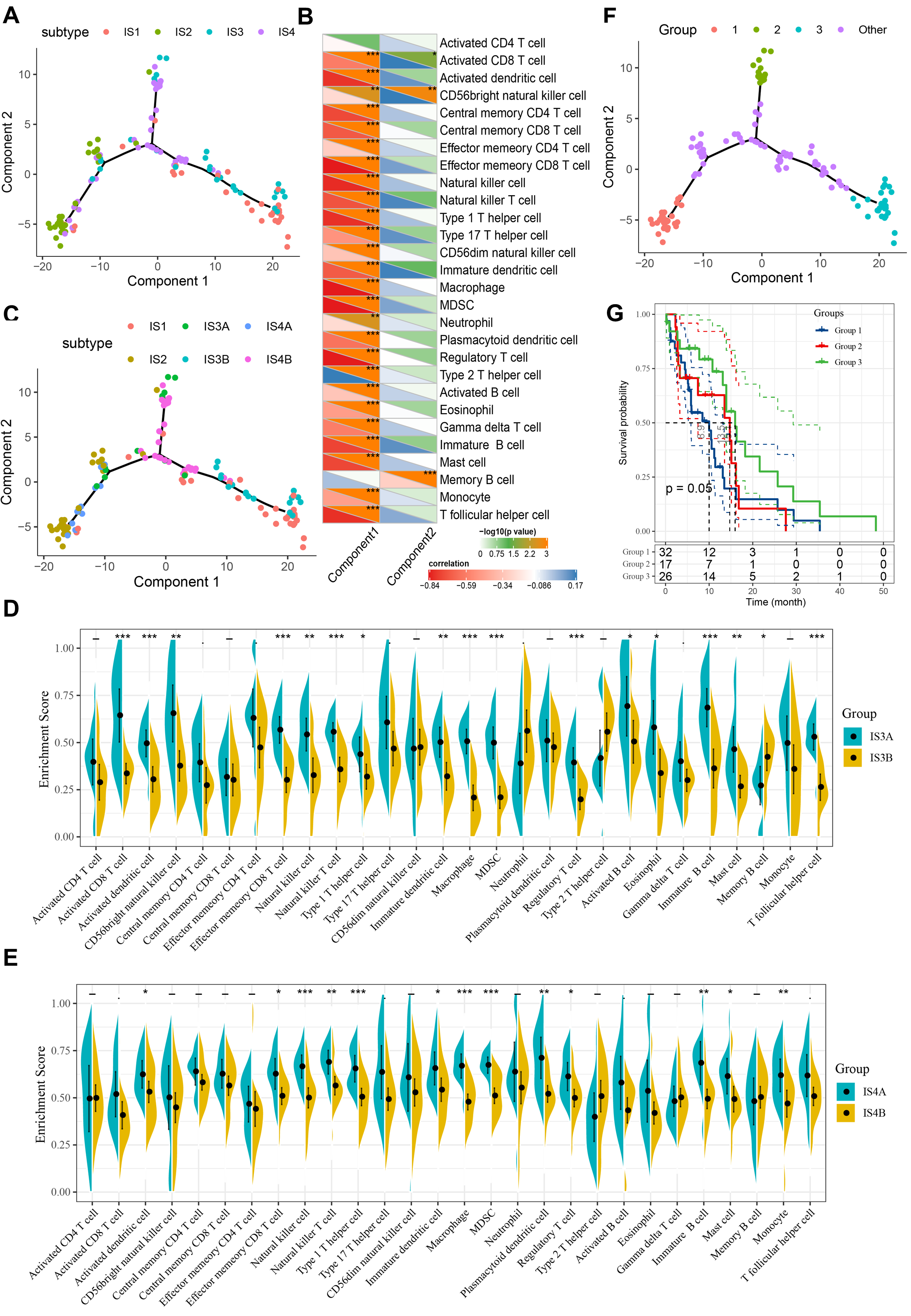


**Figure S18. The immune landscape of IDH-wildtype GBM. a** The immune landscape of IDH-wildtype GBM. Each dot represents a patient, and different colors represent different immune subtypes. The horizontal axis represents the principal component 1, and the vertical axis represents the principal component 2. **b** Correlation between principal component 1/2 and 28 immune cell enrichment scores. **c** Immune landscape of the subgroups of IDH-wildtype GBM immune subtypes. **d,e** Differences of 28 immune cells enrichment scores in the subgroups of IS3 **(d)** and IS4 **(e)**. **f,g** Immune landscape of samples from three extreme locations (**f**) and their prognostic status (**g**). - *p*≥ 0.1, ·*p* < 0.1, * *p* < 0.05, ** *p* < 0.01, *** *p* < 0.001


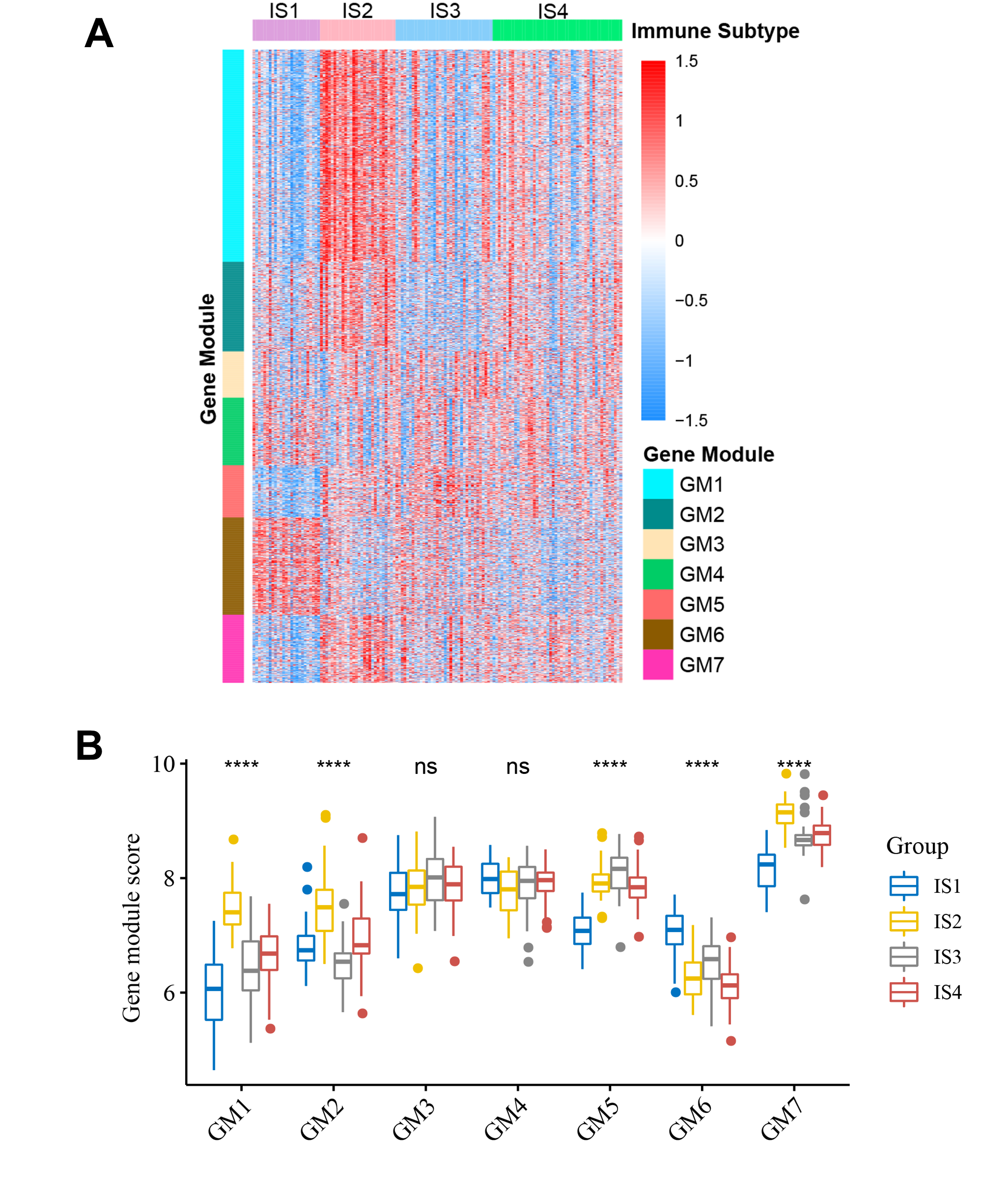


**Figure S19. Functional immune genes modules in IDH-wildtype GBM. a** Heatmap of four immune subtypes and seven gene modules in IDH-wildtype GBM. Genes are ordered based on the gene modules, and patients are arranged based on their immune subtypes. **b** Box plots of the expression patterns of seven gene modules of four immune subtypes in IDH-wildtype GBM. **** *p* < 0.0001

**Supplementary tables**

Table S1. Clinical characteristics of GBM patients in the TCGA cohort, REMBRANDT cohort and PD-1 inhibitor cohort.

| **Characteristics** | **TCGA cohort (N=143)** | **REMBRANDT cohort (N=181)** | **PD-1 inhibitor cohort (N=17)** |
| --- | --- | --- | --- |
| **Age** | 21~89 | - | 31~70 |
| ≤60 years | 77 (53.85%) | - | 9 (52.94%) |
| >60 years | 66 (46.15%) | - | 8 (47.06%) |
| **Gender** |  |  |  |
| Male | 94 (65.73%) | - | 9 (52.94%) |
| Female | 49 (34.27%) | - | 8 (47.06%) |
| **IDH1 status** |  |  |  |
| Mutant | 6 (4.20%) | - | - |
| Wild-type | 133 (93.00%) | - | - |
| NA | 4 (2.80%) | - | - |
| **MGMT status** |  |  |  |
| Methylated | 49 (34.27%) | - | - |
| Unmethylated | 62 (43.36%) | - | - |
| NA | 32 (22.37%) | - | - |
| **CIMP status** |  |  |  |
| G-CIMP | 8 (5.60%) | 8 (4.42%) | - |
| Non-G-CIMP | 135 (94.40%) | 173 (95.58%) | - |
| **Vital status** |  |  |  |
| Alive | 50 (34.97%) | 9 (4.97%) | 9 (52.94%) |
| Dead | 93 (65.03%) | 172 (95.03%) | 8 (47.06%) |
| **Subtype** |  |  |  |
| Classical | 55 (38.46%) | 63 (34.81%) | - |
| Mesenchymal | 48 (33.57%) | 59 (32.60%) | - |
| Proneural | 40 (27.97%) | 59 (32.59%) | - |

-, data unavailable; NA, nodata.

Table S4. IGP was estimated for each immune subtype in the validation cohort.

| Immune subtype | IS1 | IS2 | IS3 | IS4 |
| --- | --- | --- | --- | --- |
| IGP value | 0.737 | 0.852 | 0.774 | 0.495 |

Table S5. Functional enrichment analysis of gene modules.

| **Gene module** | **Annotation** | **Top 5 enriched biological processes** |
| --- | --- | --- |
| GM1 | T cell | GO:0042110~T cell activation  GO:0051249 ~regulation of lymphocyte activation  GO:0007159 ~leukocyte cell-cell adhesion  GO:0050867 ~positive regulation of cell activation GO:1903039~positive regulation of leukocyte cell-cell adhesion |
| GM 2 | Reactive stroma | GO:0030198~extracellular matrix organization  GO:0043062 ~extracellular structure organization  GO:0001667 ~ameboidal-type cell migration  GO:0050900 ~leukocyte migration  GO:0031589~cell-substrate adhesion |
| GM 3 | Angiogenesis | GO:1901342~regulation of vasculature development  GO:0045765~regulation of angiogenesis  GO:0001667 ~ameboidal-type cell migration  GO:0040013~negative regulation of locomotion  GO:0031589 ~cell-substrate adhesion |
| GM 4 | Cell morphogenesis | GO:0022604~regulation of cell morphogenesis  GO:0048638~regulation of developmental growth  GO:0007409 ~axonogenesis  GO:0007411~axon guidance  GO:0010769~regulation of cell morphogenesis involved in differentiation |
| GM 5 | IFN-γ | GO:0060337~type I interferon signaling pathway  GO:0051607~defense response to virus  GO:0071357~cellular response to type I interferon  GO:0060333 ~interferon-gamma-mediated signaling pathway  GO:0009615~response to virus |
| GM 6 | SMAD protein phosphorylation | GO:0010862~positive regulation of pathway-restricted SMAD protein phosphorylation  GO:0060393~regulation of pathway-restricted SMAD protein phosphorylation  GO:0060389 ~pathway-restricted SMAD protein phosphorylation  GO:0007409~axonogenesis  GO:0019932~second-messenger-mediated signaling |
| GM 7 | Antigen processing and presentation | GO:0002474~antigen processing and presentation of peptide antigen via MHC class I  GO:0002479~antigen processing and presentation of exogenous peptide antigen via MHC class I, TAP-dependent  GO:0042590~antigen processing and presentation of exogenous peptide antigen via MHC class I  GO:0002831~regulation of response to biotic stimulus  GO:0031349~positive regulation of defense response |
